# Taxonomic study on fourteen symphytognathid species from Asia (Araneae, Symphytognathidae)

**DOI:** 10.3897/zookeys.1072.67935

**Published:** 2021-11-19

**Authors:** Ya Li, Shuqiang Li, Yucheng Lin

**Affiliations:** 1 Key Laboratory of Bio-resources and Eco-environment (Ministry of Education), College of Life Sciences, Sichuan University, Chengdu, Sichuan 610064, China; 2 Institute of Zoology, Chinese Academy of Sciences, Beijing 100101, China; 3 The Sichuan Key Laboratory for Conservation Biology of Endangered Wildlife, Sichuan University, Chengdu, Sichuan 610064, China

**Keywords:** Dwarf orb-weavers, new species, new genus, new combination, China, Vietnam, Thailand, Myanmar

## Abstract

Fourteen symphytognathid species belonging to three genera are examined, including the descriptions of eight new species and two new genera from China, Vietnam, Thailand and Myanmar: *Patu* Marples, 1951: *P.catba* S. Li & Lin, **sp. nov.** (♂, Vietnam), *P.dakou* S. Li & Lin, **sp. nov.** (♂♀, China), *P.damtao* S. Li & Lin, **sp. nov.** (♂, Vietnam), *P.jiangzhou* S. Li & Lin, **sp. nov.** (♀, China), *P.jidanweishi* Miller, Griswold & Yin, 2009 (♂♀, China), *P.nagarat* S. Li & Lin, **sp. nov.** (♂♀, Thailand), *P.nigeri* Lin & S. Li, 2009 (♀, China), *P.putao* S. Li & Lin, **sp. nov.** (♀, Myanmar), *P.qiqi* Miller, Griswold & Yin, 2009 (♀, China) and *P.xiaoxiao* Miller, Griswold & Yin, 2009 (♂♀, China); *Kirinua* S. Li & Lin, **gen. nov.**: *K.maguai* S. Li & Lin, **sp. nov.** (♂♀, China) and *K.yangshuo* S. Li & Lin, **sp. nov.** (♂♀, China); *Swilda* S. Li & Lin, **gen. nov.**: *S.longtou* (Miller, Griswold & Yin, 2009), **comb. nov.** (♂♀, China) is transferred from *Crassignatha* Wunderlich, 1995 and *S.spinathoraxi* (Lin & S. Li, 2009), **comb. nov.** (♂♀, China) is transferred from *Patu*. Diagnoses, descriptions and illustrations are provided for new taxa, as well as a distribution map. The males of *P.xiaoxiao* and *S.longtou* are described for the first time. Type specimens of *P.jidanweishi*, *P.nigeri*, *P.qiqi*, *P.xiaoxiao*, *S.longtou* and *S.spinathoraxi* are re-examined and photographed. All Asian *Patu* species are revised and two species, *P.kishidai* Shinkai, 2009 and *P.bispina* Lin, Pham & S. Li, 2009, are transferred to *Crassignatha* and proposed as new combinations: *Crassignathakishidai***comb. nov.** and *C.bispina***comb. nov.** In addition, DNA barcodes and genetic distances of ten species treated in this paper were obtained to confirm identification.

## Introduction

Symphytognathidae Hickman, 1931 is a small spider family mainly distributed in tropical and subtropical regions of the Oriental and Neotropical realms. Ninety symphytognathid species in eight genera are known, of which 45 species and six genera occur in Asia (WSC 2021).

Before the current study, fourteen species from China, Colombia, Fiji, Japan, New Guinea, Samoa, Seychelles and Vietnam were assigned to *Patu* Marples, 1951. Miller et al. (2009) mentioned that *Patu* is a particularly problematic genus because of insufficient study of the copulatory organs, the scarcity of material in collections and the minute size of these animals. A complete revision of this genus is still lacking and the taxonomic placement of some doubtful species needs to be clarified.

The aim of this paper is to provide data on Asian *Patu* and related genera, including descriptions of new species and genera, based on recent collections from China, Vietnam, Thailand and Myanmar.

## Materials and methods

More than 1200 adult symphytognathid specimens were examined in a 95% ethanol solution under a Leica M205 C stereomicroscope. The digital photos were montaged using Helicon Focus 3.10 (Khmelik et al. 2006) image stacking software. Male palps and epigynes were examined and photographed after dissection. The left palp was photographed and described (if missing, the right was used). Epigynes were treated with lactic acid before being embedded in Hoyer’s Gum and placed on an ultra-thin slide to take photos of both sides of the vulva. All measurements are in millimetres. Leg measurements are given as follows: total length (femur, patella, tibia, metatarsus and tarsus).

Tissue samples were taken from the prosoma of 17 individuals of *Patu*, *Kirinua* S. Li & Lin, gen. nov. and *Swilda* S. Li & Lin, gen. nov., including five new and five known species (the abdomens and male palps were kept as vouchers). All of the molecular data were obtained from specimens collected at the type localities, although not from the type specimens themselves. A partial fragment (636 bp) of the mitochondrial gene cytochrome *c* oxidase subunit I (COI) was amplified and sequenced to calculate the genetic distances between morphologically-similar species to confirm identifications and for sex pairing.

The primers used were: LCO1490 (5’-GGTCAACAAATCATCATAAAGATATTGG-3’) and HCO2198 (5’-TAAACTTCAGGGTGACCAAAAAA TCA-3’). Raw sequences were edited and assembled using BioEdit v.7.2.5 (Hall 1999) and the uncorrected pairwise distances between species were calculated using MEGA7.0.14 (Kumar et al. 2016). Results of the genetic distance analysis are shown in Appendix Table A1.

Morphological abbreviations used in the figures are given in Table 1. New sequences, generated for this study, are available from GenBank and the accession numbers are reported in Table 2. References to figures in the cited papers are listed in lowercase (fig. or figs) and figures in this paper are noted with an initial capital (Fig. or Figs). With the exception of the types of previously-described species kept in **HNU** and **IZCAS**, all molecular vouchers are deposited in **NHMSU** in Chengdu, China and examined morphological material is deposited in **NHMSU** and **IZCAS**.

**Table 1.** List of abbreviations used in the text or figures.

Male palp		Epigyne	
**AP**	apical process on tegulum	**Atr**	atrium
**Co**	conductor	**CD**	copulatory duct
**Cy**	cymbium	**CO**	copulatory opening
**CA**	cymbial apophysis	**FD**	fertilisation duct
**CP1**	proximal cymbial process	**Pl**	parmula
**CP2**	distal cymbial process	**S**	spermatheca
**E**	embolus	**Sp**	scape
**Fe**	femur	**Somatic characters**	
**MA**	median apophysis	**ALE**	anterior lateral eyes
**Pa**	patella	**PLE**	posterior lateral eyes
**T**	tegulum	**PME**	posterior median eyes
**Ti**	tibia	**PER**	posterior eye row
**TP**	tegular process	**TS**	male clasping spines on tibia II
**Institutions**			
**HNU**	College of Life Sciences, Hunan Normal University, Changsha, China		
**IZCAS**	Institute of Zoology, Chinese Academy of Sciences, Beijing, China		
**NHMSU**	Natural History Museum of Sichuan University, Chengdu, China		

**Table 2.** GenBank accession numbers for DNA sequence data from ten symphytognathid species.

Species	Identifier	Sample	COI*	Collection localities
*Patudakou* sp. nov.	HA135	1♂	MW970248	China, Yunnan, Longling County
	HA135	1♀	MW970247	
*Patujiangzhou* sp. nov.	HA012	1♀	MW970234	China, Guangxi, Fengshan County
*Patujidanweishi*	HA119	1♂	MW970243	China, Yunnan, Fugong County
	HA119	1♀	MW970242	
*Patunagarat* sp. nov.	HA087	1♂	MW970240	Thailand, Khon Kaen Pro.
	HA087	1♀	MW970239	
*Patunigeri*	HA129	1♀	MW970246	China, Yunnan, Gongshan County
*Patuxiaoxiao*	HA123	1♂	MW970245	China, Yunnan, Lushui County
	HA123	1♀	MW970244	
*Kirinuamaguai* sp. nov.	HA008	1♀	MW970250	China, Guangxi, Fengshan County
*Kirinuayangshuo* sp. nov.	HA018	1♂ juv.	MW970236	China, Guangxi, Yangshuo County
	HA018	1♀	MW970235	
*Swildalongtou*	HA112	1♂	MW970249	China, Yunnan, Fugong County
	HA112	1♀	MW970241	
*Swildaspinathoraxi*	HA082	1♂	MW970238	China, Yunnan, Mengla County
	HA082	1♀	MW970237	

## Taxonomy

### Family Symphytognathidae Hickman, 1931

#### Genus Patu

Taxon classificationAnimaliaAraneaeSymphytognathidae

Marples, 1951

4739C1B3-B00F-5DF4-9805-32C80AD75CEC

Patu Marples, 1951: 47.
Patu Forster, 1959: 318.
Patu Forster & Platnick, 1977: 15.

##### Type species.

*Patuvitiensis* Marples, 1951 by original designation, from Fiji.

##### Diagnosis.

**Figure 1.**
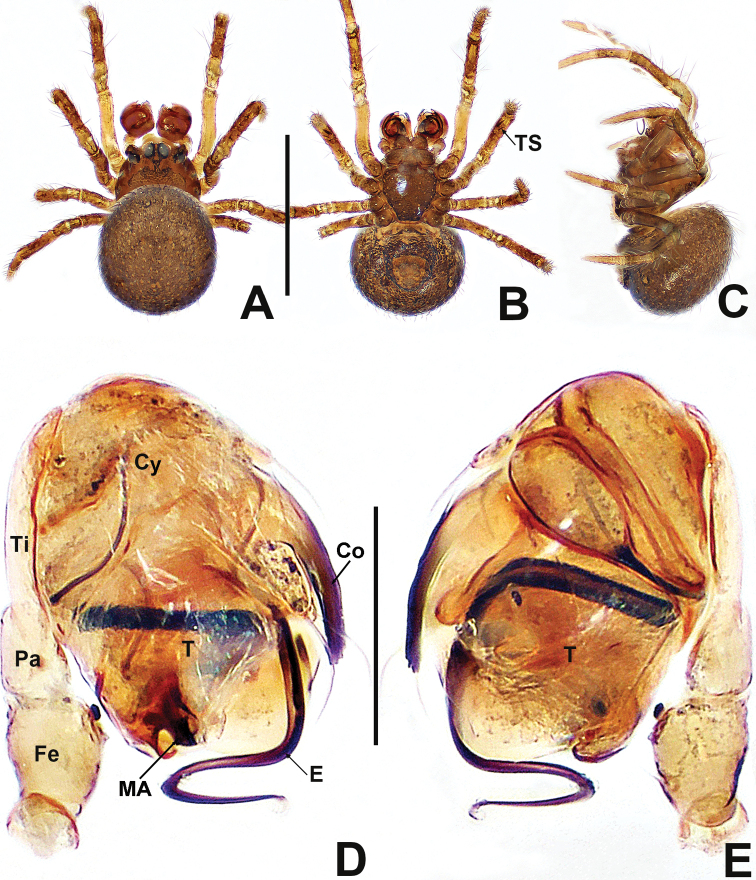
*Patucatba* sp. nov. **A** male habitus, dorsal **B** male habitus, ventral **C** male habitus, lateral **D** male palp, prolateral **E** male palp, retrolateral. Abbreviations: Co = conductor; Cy = cymbium; E = embolus; Fe = femur; MA = median apophysis; Pa = patella; T = tegulum; Ti = tibia; TS = male clasping spines on tibia II. Scale bars: 0.50 (**A–C**); 0.10 (**D, E**).

*Patu* can be distinguished from *Anapistula* Gertsch, 1941 by having 6 eyes vs. four or lacking and from *Anapogonia* Simon, 1905, tentatively placed in Symphytognathidae (Platnick and Forster 1989: 76), by the chelicerae fused at the mid-line vs. unfused. *Patu* differs from *Globignatha* Balogh & Loksa, 1968 and *Symphytognatha* Hickman, 1931 by the chelicerae fused only at mid-line vs. almost fully fused, see Lin 2019: fig. 1H. It differs from *Curimagua* Forster & Platnick, 1977 by having 6 eyes in diads and the female lacking palps (Fig. 8A and C) vs. 6 eyes in triads and female palps reduced to vestiges (Forster and Platnick 1977: figs 40 and 63). *Patu* differs from *Iardinis* Simon, 1899 (*I.martensi* Brignoli, 1978 from Nepal and *I.mussardi* Brignoli, 1980 from India) by having clasping spines on tibia II on the male, but lacking in the latter and from *Crassignatha* Wunderlich, 1995 and *Swilda* gen. nov. by lacking a latero-posterior abdominal scutum in the male and the rod-shaped or oval spermathecae (Figs 1C, 9F and 10G) vs. having an abdominal scutum and spherical spermathecae (Figs 19A, 21A and 22E; Li, Lin and Li 2020: figs 16C and 22D). *Patu* is similar to *Kirinua* gen. nov. by the absence of a latero-posterior abdominal scutum in the male and the carapace surface lacking granular or spinous ornaments in both sexes, but it can be distinguished by the male having sulci and pores on the clypeus, rather than a pair of pocket-shaped pits in the latter and the male palpal cymbium lacks accessorial structures (e.g. primary conductor, cymbial process or apophysis); females can be distinguished by having rod-shaped or oval spermathecae rather than spherical or subspherical spermathecae in the latter.

##### Description.

**Figure 2.**
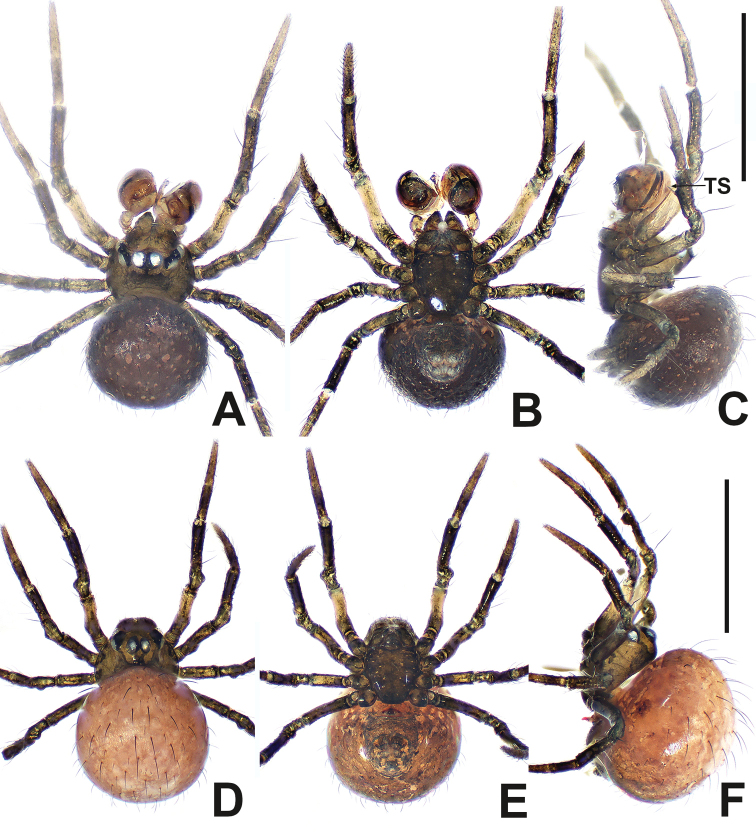
*Patudakou* sp. nov. **A** male habitus, dorsal **B** male habitus, ventral **C** male habitus, lateral **D** female habitus, dorsal **E** female habitus, ventral **F** female habitus, lateral. Abbreviation: TS = male clasping spines on tibia II. Scale bars: 0.50 (**A–F**).

Tiny, total length 0.40–0.80. Carapace round in male, pear-shaped in female dorsally, nearly triangular laterally (Figs 6A, 2D, 8A and 8D). Six eyes in 3 diads, ocular base black, AME absent, lateral eyes adjacent, cephalic part raised (Figs 2C, 2F, 8C and 8F). Clypeus concave, with modified sulci and pores (fig. 69A–D in Miller et al. 2009). Female lacking palps. Chelicerae fused at middle, with a single tooth (fig. 69E and F in Miller et al. 2009). Labium wider than long, fused to sternum (Figs 2B, 2E, 8B and 8E). Sternum heart shaped, truncated posteriorly. Male tibia II with 1–2 clasping spines subdistally (Figs 1C, 2C, 6C and 8C). Abdomen globular dorsally, subovoid laterally, without latero-posterior abdominal scutum (Figs 1C, 4C and 8C). Spinnerets without annular plates. Colulus absent.

**Figure 3.**
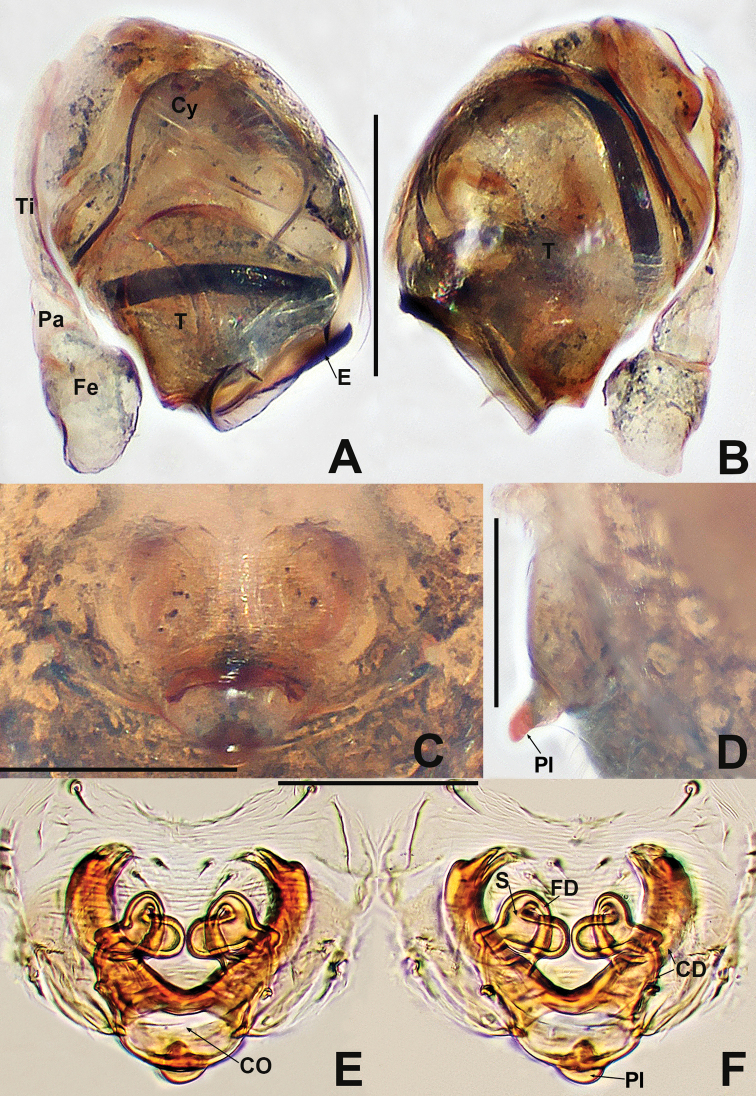
*Patudakou* sp. nov. **A** male palp, prolateral **B** male palp, retrolateral **C** epigyne, ventral **D** epigyne, lateral **E** vulva, ventral **F** vulva, dorsal. Abbreviations: CD = copulatory ducts; CO = copulatory opening; Cy = cymbium; E = embolus; FD = fertilisation ducts; Fe = femur; MA = median apophysis; Pa = patella; Pl = parmula; S = spermathecae; T = tegulum; Ti = tibia. Scale bars: 0.10 (**A–F**).

**Figure 4.**
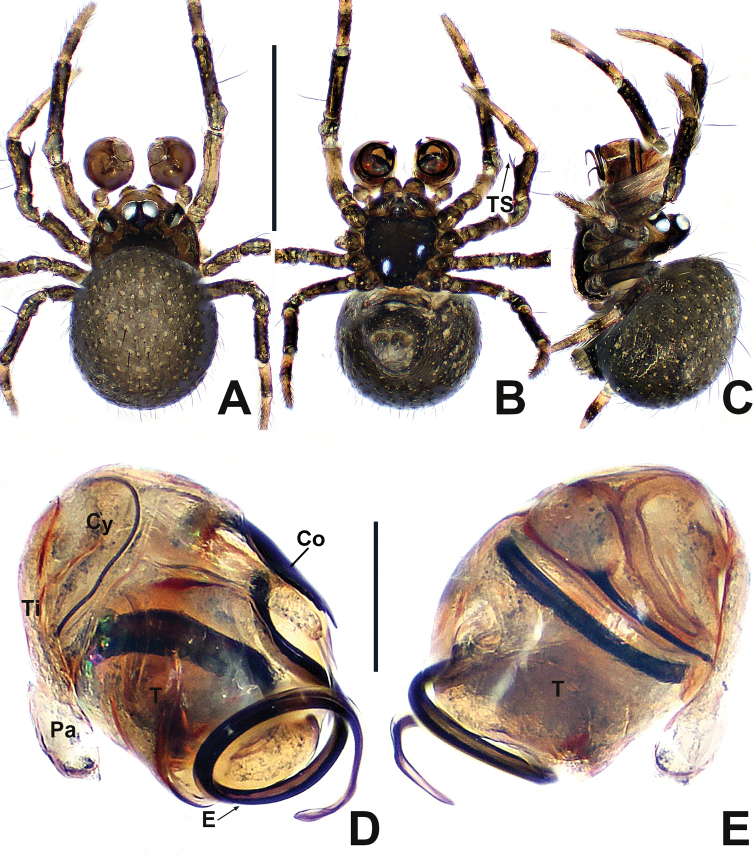
*Patudamtao* sp. nov. **A** male habitus, dorsal **B** male habitus, ventral **C** male habitus, lateral **D** male palp, prolateral **E** male left palp, retrolateral. Abbreviations: Co = conductor; Cy = cymbium; E = embolus; Pa = patella; T = tegulum; Ti = tibia; TS = male clasping spines on tibia II. Scale bars: 0.50 (**A–C**); 0.10 (**D, E**).

***Male palp*** (Figs 4D, E, 8A and B): bulb nearly ovate, large, not less than ~ ¼ size of carapace. Cymbium membranous, translucent, wrapping around bulb prolaterally, without modified teeth, processes or apophyses. Conductor usually absent (if present, long, finger-like, starting at dorsal side of bulb, close to embolic base, see Figs 1D and 4D). Tegulum cup shaped, with 1 or 2 protrusions (median apophysis and tegular process) (Figs 1D, 7A and 9A). Embolus long, slender, tubular, coiling into at least 2 loops within tegulum, distal part of embolus embedded inside bulb (Figs 3A, B, 14A and B) or extends and twists at tip of bulb (Figs 1D, E, 4D, E, 7A, B, 9A and B).

***Epigyne*** (Figs 7E, F, 9E, F, 14D and E): weakly sclerotised. Scape or parmula short, tongue-shaped or long, finger-like. Spermathecae long, ovate or kidney-shaped, separated by less than 2 lengths. Copulatory openings separated. Copulatory ducts membranous or faintly sclerotised, usually partially or completely surround spermathecae (exceptions are *P.nigeri* Lin & S. Li, 2009 and *P.qiqi*). Fertilisation ducts short, thin, typically originate from the lateral or anterior side of spermathecae.

##### Composition.

*Patucatba* sp. nov. (♂), *P.dakou* sp. nov. (♂♀), *P.damtao* sp. nov. (♂), *P.digua* Forster & Platnick, 1977 (♂♀), *P.eberhardi* Forster & Platnick, 1977 (♂♀), *P.jiangzhou* sp. nov. (♀), *P.jidanweishi* Miller, Griswold & Yin, 2009 (♂♀), *P.marplesi* Forster, 1959 (♂), *P.nagarat* sp. nov. (♂♀), *P.nigeri* (♂♀), *P.putao* sp. nov. (♀), *P.qiqi* Miller, Griswold & Yin, 2009 (♂♀), *P.saladito* Forster & Platnick, 1977 (♀), *P.samoensis* Marples, 1951 (♂♀), *P.silho* Saaristo, 1996 (♂♀), *P.vitiensis* Marples, 1951 (♂♀), *P.woodwardi* Forster, 1959 (♂♀) and *P.xiaoxiao* Miller, Griswold & Yin, 2009 (♂♀). Li, Lin and Li (2020) have previously suggested that the other two Asian species, *P.bispina* Lin, Pham & S. Li, 2009 from Vietnam and *P.kishidai* Shinkai, 2009 from Japan, do not belong to this genus and should be transferred to *Crassignatha*. Based on the above diagnosis of *Patu*, we formally propose two new combinations: *Crassignathabispina* comb. nov. and *C.kishidai* comb. nov.

##### Distribution.

China (Guangxi, Hainan, and Yunnan), Colombia, Fiji, Myanmar, New Guinea, Samoa, Seychelles, Thailand, Vietnam.

##### Remarks.

Of the male *Patu* species described here, the embolus is either embedded within the tegulum or not, the conductor is present or absent and the tegular process is present or absent. The similarities of the palps are the nearly ovate bulb and the cymbium lacking any teeth, processes or apophyses. In the females, the epigyne and vulva distinctly differ in the type, shape and size of posterior process of the epigyne (scape or parmula) and in the texture, length and course of the copulatory ducts. The similarities of the vulvae are the ovate or short, club-shaped spermathecae.

#### Patu catba

Taxon classificationAnimaliaAraneaeSymphytognathidae

S. Li & Lin sp. nov.

5F15398C-0954-536E-A91B-9FC6613E300C

http://zoobank.org/2CB7474E-ACD6-410C-8344-E507BDC06219

Figures 1 , 23

##### Type material.

***Holotype*** ♂ (IZCAS-Ar 41036) **Vietnam**: Cat Ba National Park, Hai Phong Province, in leaf litter of natural forest (20.80133°N, 107.00353°E; 116 m alt.), 23.IX.2007, D. Pham leg.

##### Etymology.

The specific epithet derives from the type locality; noun in apposition.

##### Diagnosis.

This new species differs from other *Patu* species with the exception of *P.damtao* sp. nov. by having a long, sclerotised conductor dorsally on the bulb (Figs 1D and 4D). It can be distinguished from *P.damtao* sp. nov. by the blunt conductor swollen basally, the shorter embolus forming no more than one loop and the presence of a median apophysis (Fig. 1D and E) vs. a sharp conductor constricted basally, a longer embolus forming more than one loop and the absence of a median apophysis (Fig. 4D and E).

##### Description.

**Male** (IZCAS-Ar 41036). Total length 0.56. Carapace 0.24 long, 0.28 wide, 0.32 high. Clypeus 0.14 high. Sternum 0.16 long, 0.16 wide. Abdomen 0.40 long, 0.36 wide, 0.40 high. Length of legs: I 0.74 (0.20, 0.10, 0.16, 0.12, 0.16); II 0.66 (0.20, 0.10, 0.12, 0.10, 0.14); III 0.50 (0.12, 0.08, 0.08, 0.08, 0.14); IV 0.56 (0.12, 0.10, 0.12, 0.10, 0.12).

**Somatic characters** (Fig. 1A–C). ***Colouration***: carapace brown, nut brown at margins, ocular base black, thoracic region reticulated medially. Chelicerae, endites and labium dark brownish. Sternum nut brown. Legs brownish to dark yellow, except femur I yellow. Abdomen brown and grey, with dense, light dots. ***Prosoma***: carapace wider than long, nearly pyriform. Eyes with ocular tubercles, PME > ALE > PLE, ALE protruded, PER recurved. Sternum slightly swollen, smooth and glossy, sparsely punctate. ***Legs***: with 1 dorso-distal seta on each patella and 1 seta on each tibia sub-proximally. Tibia II with 2 subdisto-ventral clasping spines. ***Opisthosoma***: dorsally rounded, laterally nearly ovoid. Spinnerets brown, darker at edges.

***Palp*** (Fig. 1D and E): large, ~ ⅓ size of carapace. Femur slightly swollen, patella ~ ½ length of tibia, tibia flat. Cymbium translucent, membranous, surrounds bulb prolaterally, with a finger-like extension, with 2 distal, long setae. Bulb subovate. Tegulum smooth. Median apophysis short, not extending beyond apex of bulb. Conductor long, finger-like, sclerotised, basally swollen, distally blunt. Embolus ca. 2× length of conductor, ribbon-like, middle portion embedded in tegulum, distal portion looped at the apex of bulb.

**Female.** Unknown.

##### Distribution.

Vietnam (Fig. 23).

#### Patu dakou

Taxon classificationAnimaliaAraneaeSymphytognathidae

S. Li & Lin sp. nov.

4B95E1F1-6C7D-5BAB-9791-600E173269D0

http://zoobank.org/6F994EA1-BBCC-4226-9C71-1ECF912D901B

Figures 2 , 3 , 23

##### Type material.

***Holotype*** ♂ (NHMSU Ar 132) and ***paratypes*** 2♂ 7♀ (NHMSU Ar 133–141) **China**: Yunnan Province, Longling County, Zhen’an Township, Bangbie Village at stream at 6.8 km on S317 Road, shaded embankments along stream, dusting webs in understorey (24.81333°N, 98.83280°E; 1560 m alt.), 22.VIII.2018, Y. Lin et al. leg.; 1♂ (NHMSU-HA135) and 1♀ (NHMSU-HA135) used for sequencing, GenBank: MW970248 and MW970247, same data as for preceding.

##### Etymology.

Formed from the Chinese word (dà kŏu), referring to the large copulatory opening of the epigyne (Fig. 3C and E); noun.

##### Diagnosis.

The new species differs from other congeners with the exception of *P.nigeri* by the embolus completely encased in the tegulum, the knob-shaped parmula and the proximal position of the copulatory ducts forming a pair of horn-like structures (Fig. 3A–F). The male of *P.dakou* sp. nov. is similar to that of *P.nigeri*, but it can be distinguished by the more basal position of the embolus (Fig. 3A and B vs. fig. 4A and B in Lin and Li 2009). The female is similar to that of *P.nagarat* sp. nov. in the configuration of the vulva, but it differs by the nearly adjacent spermathecae, the knob-shaped parmula and the fertilisation ducts originating from the anterior side of the spermathecae vs. separated spermathecae, a triangular parmula and the fertilisation ducts originating laterally on the spermathecae (Fig. 3D–F vs. Fig. 9D–F).

##### Description.

**Male** (NHMSU Ar 132). Total length 0.56. Carapace 0.28 long, 0.28 wide, 0.28 high. Clypeus 0.08 high. Sternum 0.20 long, 0.20 wide. Abdomen 0.36 long, 0.40 wide, 0.36 high. Length of legs: I 0.80 (0.20, 0.06, 0.24, 0.12, 0.18); II 0.64 (0.12, 0.06, 0.16, 0.14, 0.16); III 0.46 (0.12, 0.06, 0.10, 0.08, 0.10); IV 0.58 (0.16, 0.10, 0.12, 0.08, 0.12).

**Somatic characters** (Fig. 2A–C). ***Colouration***: carapace dark grey, darker on thoracic margin and centre. Chelicerae, endites and labium black. Sternum black. Legs light brown, with black pigmentation. Abdomen charcoal grey, dorsally lighter than ventrally, with irregular light spots. ***Prosoma***: carapace as long as wide, dorsally rounded, laterally conical. ALE protruded, PER straight. Chelicerae with an anterior small hump (Fig. 2B). Labium semi-lunar. Sternum flat, smooth. ***Legs***: Each patella with a long disto-dorsal seta. Tibia II with 1 ventral clasping spine sub-distally. ***Opisthosoma***: dorsally rounded, laterally oval, covered with long, sparse, black setae. Spinnerets apically pale grey.

***Palp*** (Fig. 3A and B): large, ~ ½ size of carapace. Femur equal to 1.5× width of patella, patella short, ca. half of tibial length, tibia flat. Cymbial distal extension with a few long setae. Bulb nearly ovoid, anteriorly flat. Tegulum broad, smooth. Embolus originates retrolaterally, entirely encased in tegulum, coiled into ca. 3 loops. Sperm duct convoluted throughout. Embolic tip looped at apex of bulb.

**Female** (NHMSU Ar 133). Total length 0.64. Carapace 0.28 long, 0.28 wide, 0.24 high. Clypeus 0.10 high. Sternum 0.20 long, 0.20 wide. Abdomen 0.48 long, 0.48 wide, 0.48 high. Length of legs: I 0.68 (0.16, 0.10, 0.14, 0.12, 0.16); II 0.60 (0.12, 0.10, 0.12, 0.12, 0.14); III 0.50 (0.12, 0.10, 0.08, 0.08, 0.12); IV 0.58 (0.18, 0.10, 0.08, 0.08, 0.14).

**Somatic characters** (Fig. 2D–F). ***Colouration***: prosoma same as in male, opisthosoma light, ventrally darker than dorsally, post-gaster region and area around spinnerets black. ***Prosoma***: carapace round. Cephalic region lower than in male. PER slightly procurved. Mouthparts and sternum as in male, except longer labium. ***Legs***: as in male. ***Opisthosoma***: dorsally rounded, laterally ovate, covered with sparse, long, black setae. Spinnerets dark grey.

***Epigyne*** (Fig. 3C–F): internal structures faintly visible via cuticle. Parmula knob-shaped, protruded, distally sclerotised. Copulatory opening large, oval. Copulatory duct arising from the ventral base of parmula, its proximal part forming a pair of sclerotised, broad, horn-like structures at both sides of spermathecae. Spermathecae shorter than width of copulatory opening, claviform, nearly touching. Fertilisation ducts start at the anterolateral margin of spermathecae and curve downwards to centre of vulva.

##### Distribution.

China (Yunnan) (Fig. 23).

#### Patu damtao

Taxon classificationAnimaliaAraneaeSymphytognathidae

S. Li & Lin sp. nov.

B9A01358-5495-58D0-9283-9B77A270129C

http://zoobank.org/8D9961F6-2B24-4233-8F45-77D043BFB1F7

Figures 4 , 23

##### Type material.

***Holotype*** ♂ (IZCAS-Ar 41037) **Vietnam**: Dam Tao National Park (21.47200°N, 105.63644°E; 1023 m alt.), 31.X.2012, H. Zhao and Z. Chen leg.

##### Etymology.

The specific epithet derives from the name of the type locality; noun in apposition.

##### Diagnosis.

*Patudamtao* sp. nov. can be distinguished from other congeners, with the exception of *P.catba* sp. nov., by having a conductor and lacking a tegular process (Fig. 4D). It is similar to *P.catba* sp. nov. in the shape of the male palp, but it differs by lacking a median apophysis, the embolus has more coils and is 4× the length of the conductor vs. having a median apophysis and an embolus with fewer coils that is ca. 2× the length of the conductor (Figs 4D and E vs. 1D and E).

##### Description.

**Male** (IZCAS-Ar 41037). Total length 0.60. Carapace 0.24 long, 0.28 wide, 0.32 high. Clypeus 0.16 high. Sternum 0.20 long, 0.20 wide. Abdomen 0.40 long, 0.36 wide, 0.44 high. Length of legs: I 0.84 (0.24, 0.10, 0.18, 0.14, 0.18); II 0.68 (0.20, 0.10, 0.12, 0.10, 0.16); III 0.52 (0.12, 0.08, 0.10, 0.10, 0.12); IV 0.64 (0.16, 0.10, 0.14, 0.10, 0.14).

**Somatic characters** (Fig. 4A–C). ***Colouration***: carapace dark, darker in thoracic centre, clypeus light grey. Chelicerae, endites and labium dark. Sternum black. Legs dim yellow, with black pigmentation, tibia darkest. Abdomen charcoal grey, dorsally lighter, covered with light dots. ***Prosoma***: carapace wider than long, laterally triangular. Eyes with ocular mound, PME > ALE > PLE, ALE protruded, PER recurved. Chelicerae anteriorly plump. Labium semi-lunar. Sternum smooth, slightly plump. ***Legs***: densely covered with bristles on tibia, metatarsi and tarsi. Patella with 1 seta disto-dorsally. Tibia with 1 subproximal seta dorsally. Tibia II with 2 adnate clasping spines. ***Opisthosoma***: dorsally globular, laterally ovoid. Spinnerets darkish.

***Palp*** (Fig. 4D and E): relatively large, ~ ½ size of carapace. Patella short, about half of tibial length. Tibia flat and lamellar. Cymbium translucent, surrounding the bulb prolaterally. Bulb elongate ovoid. Tegulum smooth, cup-shaped, apically truncated. Conductor strongly sclerotised, long, spatulate, ~ ¼ length of embolus, protruded from dorsal base of tegulum, basally narrow and distally sharp. Embolus long, ca. 4× length of conductor, ribbon shaped, protruded below the conductor, coiled into ~ 1¼ loops at the apex of tegulum.

**Female.** Unknown.

##### Distribution.

Vietnam (Fig. 23).

#### Patu jiangzhou

Taxon classificationAnimaliaAraneaeSymphytognathidae

S. Li & Lin sp. nov.

6D87868D-6FA1-54FC-AE51-27BCB2842C9B

http://zoobank.org/7EB5BAB4-6C46-48F3-8B55-CBA0B67850F9

Figures 5 , 23

##### Type material.

***Holotype*** ♀ (IZCAS-Ar 41038) **China**: Guangxi Zhuang Autonomous Region, Hechi City, Fengshan County, Jiangzhou Township, underground gallery (a limestone cave) (24.33144°N, 106.98716°E; 449 m alt.), 25.III.2015, Y. Li and Z. Chen leg.; 1♀ (NHMSU-HA012) used for sequencing, GenBank: MW970234, same data as preceding.

##### Etymology.

The specific epithet derives from the name of the type locality; noun in apposition.

##### Diagnosis.

**Figure 5.**
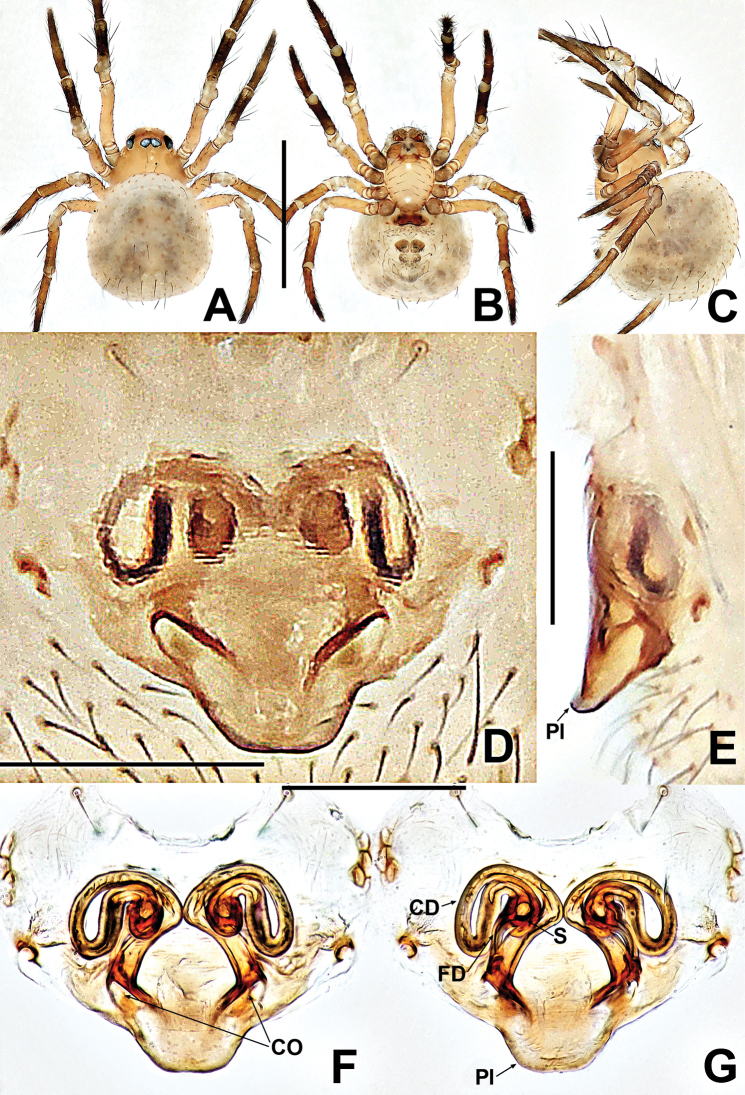
*Patujiangzhou* sp. nov. **A** female habitus, dorsal **B** female habitus, ventral **C** female habitus, lateral **D** epigyne, ventral **E** epigyne, lateral **F** vulva, ventral **G** vulva, dorsal. Abbreviations: CD = copulatory ducts; CO = copulatory opening; FD = fertilisation ducts; Pl = parmula; S = spermathecae. Scale bars: 0.50 (**A–C**); 0.10 (**D–G**).

This new species is similar to *P.putao* sp. nov. and *P.woodwardi* in the configuration of the vulva, but it differs by the smaller spermathecae that are separated by their width, the shorter copulatory ducts and the larger, trumpet-shaped copulatory openings (Fig. 5D and F–G) vs. the larger, adjacent spermathecae, the longer copulatory ducts and the smaller, circular copulatory openings (cf. Fig. 11D and F–G and fig. 123 in Forster 1959).

##### Description.

**Female** (IZCAS-Ar 41038). Total length 0.60. Carapace 0.24 long, 0.24 wide, 0.24 high. Clypeus 0.10 high. Sternum 0.16 long, 0.16 wide. Abdomen 0.44 long, 0.44 wide, 0.44 high. Length of legs: I 0.80 (0.24, 0.12, 0.16, 0.12, 0.16); II 0.74 (0.22, 0.12, 0.14, 0.10, 0.16); III 0.60 (0.14, 0.10, 0.12, 0.10, 0.14); IV 0.76 (0.22, 0.10, 0.16, 0.12, 0.16).

**Somatic characters** (Fig. 5A–C). ***Colouration***: carapace yellow. Mouthparts light brown. Sternum pale yellow. Legs light brown, tibia, metatarsi and tarsi dark grey. Abdomen pale. Spinnerets light brown. ***Prosoma***: carapace smooth, as long as wide and high, dorsally pear-shaped. Cephalic part moderately raised. ALE > PME > PLE. PER straight, lateral eyes adjacent, PME contiguous. Chelicerae stubby, with sparse, short setae anteriorly. Endites nearly quadrilateral. Labium rectangular, wider than long. Sternum faintly plump, smooth, with sparse setae. ***Legs***: 1 long disto-dorsal seta on all patella; tibia I and II with 3 dorsal setae and 1 on tibia III and IV; dense, thin setae on tibia, metatarsi and tarsi. ***Opisthosoma***: almost globose, cuticle modified by sparse, long setae and faintly ossified dots. Spinnerets brown, anterior spinnerets more fuscous than posterior spinnerets.

***Epigyne*** (Fig. 5D–G): distinctly sclerotised, internal structures faintly visible via the cuticle. Parmula tongue-shaped, wider than long, slightly protruded. Copulatory openings trumpet-shaped, located at basal side of parmula bilaterally. Spermathecae small, ovoid, separated by ca. 1.5× their diameter. Copulatory ducts long, twisted four times before connecting with the anterior margin of spermathecae. Fertilisation ducts shorter and thinner than copulatory ducts, originate at posterolateral margin of spermathecae, slightly bent and extended downwards, parallel to proximal part of copulatory ducts.

**Male.** Unknown.

##### Distribution.

China (Guangxi) (Fig. 23).

#### Patu jidanweishi

Taxon classificationAnimaliaAraneaeSymphytognathidae

Miller, Griswold & Yin, 2009

1CD9FEC3-13A3-5240-938E-C58ABB858E74

Figures 6 , 7 , 23

Patu jidanweishi Miller, Griswold & Yin, 2009: 64, figs 65A–E, 66A, B, 67A–D, 68A–F, 69A–F, 70A–F and 71A–F (♂♀).

##### Type materials examined.

***Holotype*** ♂ (CASENT 9029293, HNU) and ***paratypes*** 1♀ (CASENT 9022328, HNU) **China**: Yunnan Province, Lushui County, Pianma Township, Changyanhe, 9.3 km ESE Pianma, mixed broadleaf deciduous and evergreen forest, Winkler extraction of sifted leaf litter (25.99363°N, 98.66651°E; 2470 m alt.), 12.V.2005, C. Griswold, D. Kavanaugh and K. Guo leg.; 1♂ 1♀ (CASENT 9019863, HNU): Yunnan Province, Gaoligongshan, 0.4 km SSE Shibali forest station, dusting webs in understorey of good forest (27.16337°N, 98.78208°E; 2475 m alt.), 5.V.2004, C. Griswold leg.; 1♂ 1♀ (CASENT 9020650, HNU), 1♂ 1♀ (CASENT 9019876, HNU), 1♂ 2♀ 1 juv. (CASENT 9024143, HNU): Yunnan Province, Gaoligongshan, 0.5 km radius of Shibali forest station, dusting webs in forest (27.16519°N, 98.77891°E; 2525 m alt.), 1–9.V.2004, C. Griswold leg.; 2♂ 1♀ (CASENT 9020351, HNU): Yunnan Province, Gaoligongshan, Shibali forest station, good forest, pitfall traps (27.16636°N, 98.77667°E; 2563 m alt.), 3–11.V.2004, C. Griswold and D. Kavanaugh leg.; 4♂ 4♀ (CASENT 9000375, HNU), 14♀ (CASENT 9000373, HNU), 2♂ 10♀ 1 juv. (CASENT 9000371, HNU), 1♀ (CASENT 9000369, HNU), 1♀ (CASENT 9023115, HNU): Yunnan Province, Gaoligongshan, Nujiang Prefecture, Nujiang State Nature Reserve, Qiqihe, 9.9 km W of Gongshan (27.715°N, 98.565°E; 2000 m alt.), 9–14.VII.2000, H. Yan et al. leg.

##### Other material examined.

5♂ 60♀ (NHMSU-HA119) **China**: Yunnan Province, Lushui County, Pianma Township, Changyanhe River, 9.3 km ESE Pianma, mixed broadleaf deciduous and evergreen forest (25.99363°N, 98.66651°E; 2470 m alt.), 10.VIII.2018, Y. Lin et al. leg.; 1♂ (NHMSU-HA119) and 1♀ (NHMSU-HA119) used for sequencing, GenBank: MW970243 and MW970242, same data as for preceding; 8♂ 34♀ (NHMSU-HA120) **China**: Yunnan Province, Nujiang Prefecture, Gaoligong Mt. Nature Reserve, Qiqihe (27.71500°N, 98.56500°E; 2000 m alt.),17.VIII.2018, Y. Lin et al. leg.; 14♂ 86♀ (NHMSU-HA121) **China**: Yunnan Province, Fugong County, along the road from Shiyueliang Town to Shibali Village, native forest of mountainside (27.15546°N, 98.80573°E; 2193 m alt.), 19.VIII.2018, Y. Lin et al. leg.

##### Diagnosis.

This species differs from other congeners, except for *P.nagarat* sp. nov., by lacking a median apophysis and a conductor and having a tegular process and a long scape (Fig. 7A, B and F). It is similar to *P.nagarat* in the shape of the bulb and the configuration of the vulva, but it can be distinguished by the lack of a median apophysis, a tegular process that is shaped like the head of a sparrow (Fig. 7B) and by the rugose, finger-like scape and the more widely separated spermathecae (Fig. 7A–F) vs. having a median apophysis and a pyramidal tegular process, a broader, triangular parmula and spermathecae are closer (Fig. 9A–F).

##### Description.

**Figure 6.**
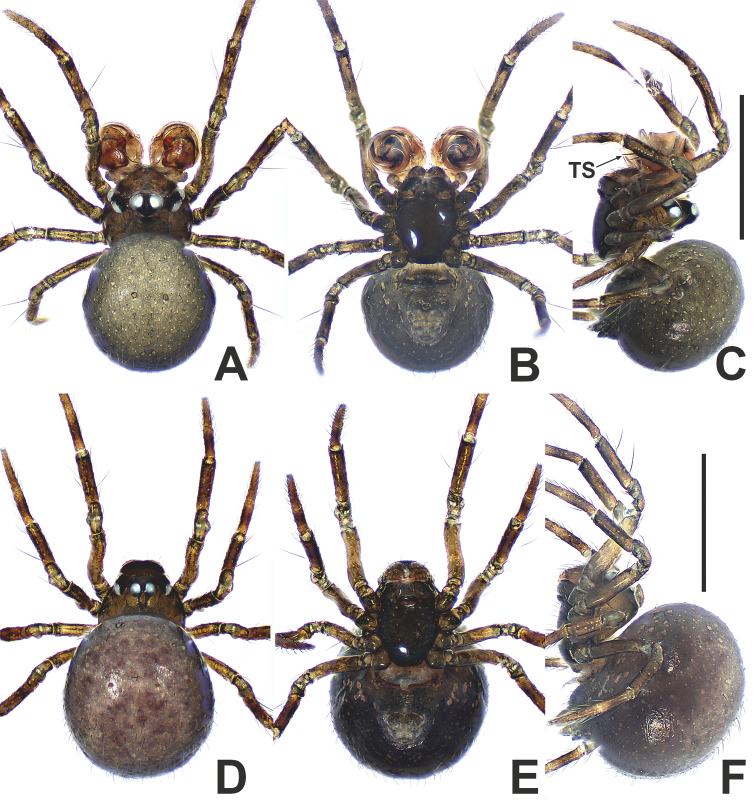
*Patujidanweishi***A** male habitus, dorsal **B** male habitus, ventral **C** male habitus, lateral **D** female habitus, dorsal **E** female habitus, ventral **F** female habitus, lateral. Abbreviation: TS = male clasping spines on tibia II. Scale bars: 0.50 (**A–F**).

**Figure 7.**
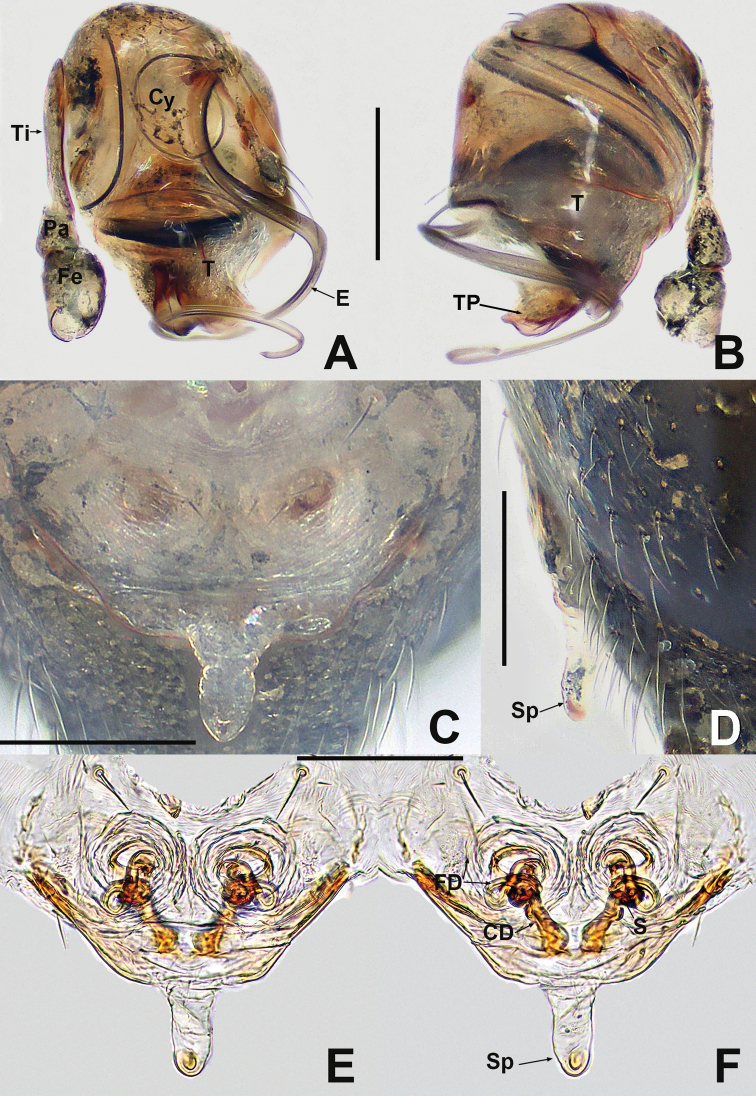
*Patujidanweishi***A** male palp, prolateral **B** male palp, retrolateral **C** epigyne, ventral **D** epigyne, lateral **E** vulva, ventral **F** vulva, dorsal. Abbreviations: CD = copulatory ducts; Cy = cymbium; E = embolus; FD = fertilisation ducts; Fe = femur; Pa = patella; S = spermathecae; Sp = scape; T = tegulum; Ti = tibia; TP = tegular process. Scale bars: 0.10 (**A–F**).

See Figs 6A–F and 7A–F and Miller et al. (2009).

##### Distribution.

China (Yunnan) (Fig. 23).

#### Patu nagarat

Taxon classificationAnimaliaAraneaeSymphytognathidae

S. Li & Lin sp. nov.

B27424FB-EE17-53AB-A472-47F23D2259D0

http://zoobank.org/91389FD1-6FEA-4E6D-A256-08765F7EA895

Figures 8 , 9 , 23

##### Type material.

***Holotype*** ♂ (IZCAS-Ar 41039) and ***paratypes*** 5♀ (IZCAS-Ar 41040~41044) **Thailand**: Khon Kaen Province, Chum Phae District, Nanoog Toom Subdistrict, Nagarat Cave (16.81402°N, 101.95663°E; 531 m alt.), 30.IX.2016, H. Zhao et al. leg.; 1♂ 3♀ (NHMSU-HA087), same data as holotype; 1♂ (NHMSU-HA087) and 1♀ (NHMSU-HA087) used for sequencing, GenBank: MW970240 and MW970239, same data as for preceding.

##### Etymology.

The specific epithet derives from the type locality; noun in apposition.

##### Diagnosis.

The male of *P.nagarat* sp. nov. can be distinguished from that of other congeners by having a bifurcate, sclerotised median apophysis and a pyramidal tegular process and lacking a conductor (Fig. 9A and B) vs. lacking a median apophysis (or if present, it is not furcate) and/or having a conductor (Figs 1D, 4D, 7A and 14A). The female is similar to that of *P.jidanweishi* in the configuration of the vulva, but it differs by having a triangular parmula and the spermathecae are closer together, rather than a finger-like scape and more widely separated spermathecae (cf. Figs 9C–F and 7C–F).

##### Description.

**Male** (IZCAS-Ar 41039). Total length 0.60. Carapace 0.24 long, 0.28 wide, 0.32 high. Clypeus 0.14 high. Sternum 0.20 long, 0.20 wide. Abdomen 0.44 long, 0.44 wide, 0.48 high. Length of legs: I 1.06 (0.32, 0.12, 0.22, 0.16, 0.24); II 0.92 (0.26, 0.12, 0.18, 0.14, 0.22); III 0.70 (0.22, 0.10, 0.12, 0.12, 0.14); IV 0.80 (0.26, 0.10, 0.16, 0.12, 0.16).

**Figure 8.**
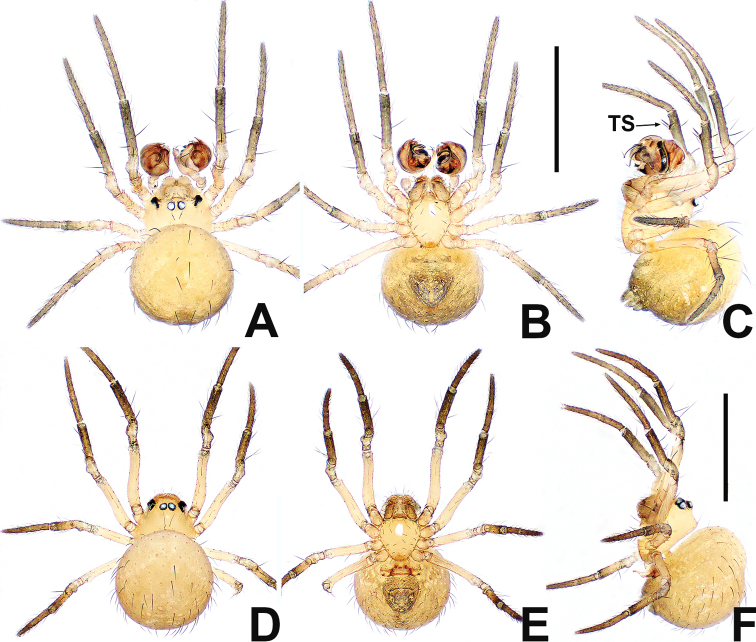
*Patunagarat* sp. nov. **A** male habitus, dorsal **B** male habitus, ventral **C** male habitus, lateral **D** female habitus, dorsal **E** female habitus, ventral **F** female habitus, lateral. Abbreviation: TS = male clasping spines on tibia II. Scale bars: 0.50 (**A–F**).

**Somatic characters** (Fig. 8A–C). ***Colouration***: body pale yellow, opisthosoma darker than prosoma, slightly grey on abdominal ventre and posterior. Leg colour a gradient, pale from femora and patella, darkening distally to dark greyish. ***Prosoma***: carapace wider than long, dorsally oval. Eyes subequal in size. ALE protruded, PER straight, PME separated by ~ ⅓ their diameter. Cephalic part with 2 setae apically, vertical anteriorly, sloped posteriorly. Chelicerae anterior surface flat. Labium semi-circular. Sternum slightly plump, smooth, with a few setae. ***Legs***: patella with 1 long disto-dorsal seta, tibia with 1 proximal and 1 mesal long dorsal seta. Tibia II with 2 ventral clasping spines subdistally, 1 thick and 1 thin (Fig. 8C). ***Opisthosoma***: globular cuticle with sparse, long, black setae. Spinnerets grey.

**Figure 9.**
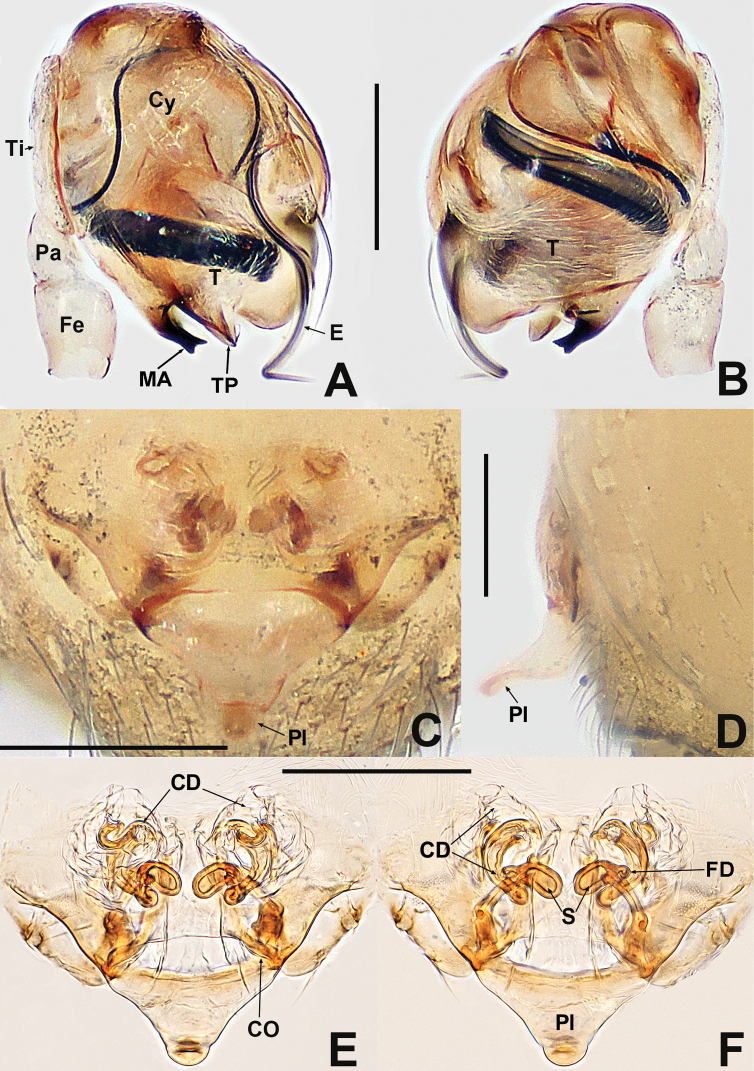
*Patunagarat* sp. nov. **A** male palp, prolateral **B** male palp, retrolateral **C** epigyne, ventral **D** epigyne, lateral **E** vulva, ventral **F** vulva, dorsal. Abbreviations: CD = copulatory ducts; CO = copulatory opening; Cy = cymbium; E = embolus; FD = fertilisation ducts; Fe = femur; MA = median apophysis; Pa = patella; Pl = parmula; S = spermathecae; T = tegulum; Ti = tibia; TP = tegular process. Scale bars: 0.10 (**A–F**).

***Palp*** (Fig. 9A and B): relatively large, ~ ½ of carapace size. Femur swollen, nearly as wide as long. Patella short, narrower than femur. Tibia flat and lamellar, length equal to ~ 2× patella. Cymbium wrapping around bulb prolaterally and ventrally, its distal extension forming triangular lamina, with 2 long setae distally. Tegulum broad, rugose, with pyramidal process. Median apophysis strongly sclerotised, bifurcate distally. Sperm duct thin, faintly visible. Embolus long, slender, with a circuitous course in basal haematodocha and tegulum. Embolus filiform, protrudes from under cymbial extension, snaking to apex of tegulum.

**Female** (IZCAS-Ar 41040). Total length 0.64. Carapace 0.32 long, 0.28 wide, 0.28 high. Clypeus 0.10 high. Sternum 0.20 long, 0.20 wide. Abdomen 0.44 long, 0.44 wide, 0.44 high. Length of legs: I 0.90 (0.28, 0.12, 0.18, 0.14, 0.18); II 0.86 (0.26, 0.12, 0.16, 0.12, 0.20); III 0.66 (0.18, 0.10, 0.10, 0.10, 0.18); IV 0.82 (0.28, 0.12, 0.14, 0.10, 0.18).

**Somatic characters** (Fig. 8D–F). ***Colouration***: same as in male. ***Prosoma***: carapace ovate dorsally. Ocular area slightly more anterior than in male. Cephalic part slightly lower than in male. ***Legs***: colour of tibia, metatarsi and tarsi darker than in male. ***Opisthosoma***: same as in male.

***Epigyne*** (Fig. 9C–F): weakly sclerotised, with a few setae medially, internal structures of vulva faintly visible via the cuticle. Parmula large, triangular, protruded ventrally. Copulatory openings located on the bilateral corners of parmula base. Spermathecae oval, distally tilted slightly downwards. Copulatory ducts mostly membranous and rugose. Proximal portion of copulatory ducts weakly sclerotised, originating at ventrolateral corners of parmula base, distal portion connected to the posterolateral margin of spermathecae. Fertilisation ducts short, starting at the anterolateral margin of spermathecae.

##### Distribution.

Thailand (Fig. 23).

#### Patu nigeri

Taxon classificationAnimaliaAraneaeSymphytognathidae

Lin & S. Li, 2009

AD48A0C9-703E-52A8-A1AF-E2D9C6783620

Patu nigeri Lin & Li, 2009: 50, figs 3A, B, 4A, B, 5A–F, 6A and B (♂♀).

##### Type material.

***Holotype*** ♂ (IZCAS) and ***paratypes*** 2♂ 6♀ (IZCAS) **China**: Yunnan Province, Mengla County, Menglun Town, Xishuangbanna Tropical Botanical Garden (21.91667°N, 101.26667°E; 556 m alt.), 19–26.III.2007, G. Zheng leg.

##### Other material examined.

1♀ (NHMSU-HA058) **China**: Yunnan Province, Mengla County, Menglun Town, Xishuangbanna Tropical Botanic Garden, Rubber-Tea plantation (21.92585°N, 101.28205°E; 561 m alt.), 10–20.VI.2007, G. Zheng leg.; 1♀ (NHMSU-HA129) **China**: Yunnan Province, Gongshan County, Dulongjiang Township, Langwanduo Village, mid-mountain forest (27.70345°N, 98.35133°E; 1473 m alt.), 15.VIII.2018, Y. Lin et al. leg.; 1♀ (NHMSU-HA129) used for sequencing, GenBank: MW970246, same data as preceding.

##### Diagnosis.

**Figure 10.**
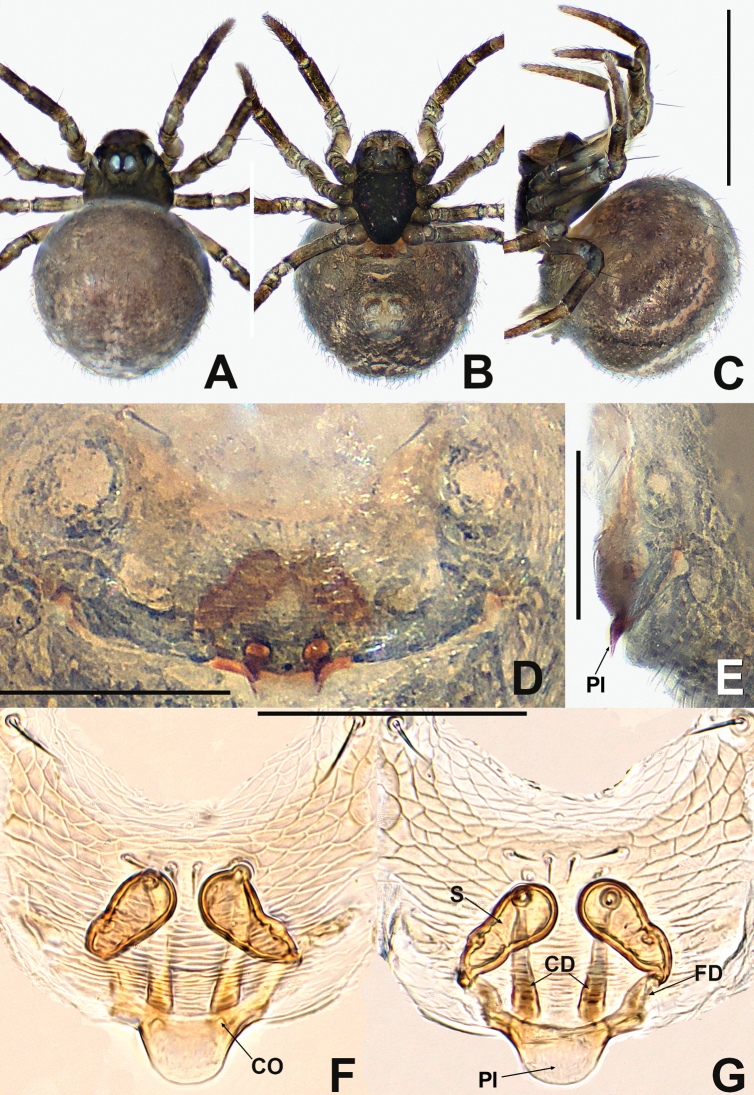
*Patunigeri***A** female habitus, dorsal **B** female habitus, ventral **C** female habitus, lateral **D** epigyne, ventral **E** epigyne, lateral **F** vulva, ventral **G** vulva, dorsal. Abbreviations: CD = copulatory ducts; CO = copulatory opening; FD = fertilisation ducts; Pl = parmula; S = spermathecae. Scale bars: 0.50 (**A–C**); 0.10 (**D–G**).

**Figure 11.**
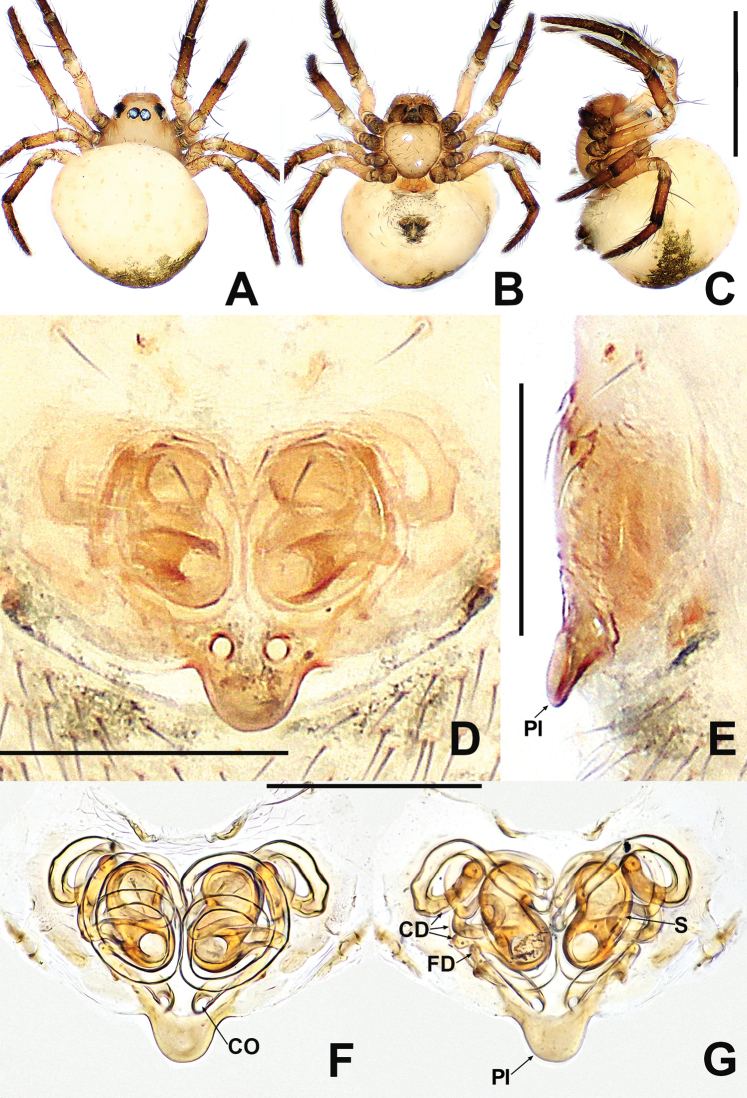
*Patuputao* sp. nov. **A** female habitus, dorsal **B** female habitus, ventral **C** female habitus, lateral **D** epigyne, ventral **E** epigyne, lateral **F** vulva, ventral **G** vulva, dorsal. Abbreviations: CD = copulatory ducts; CO = copulatory opening; FD = fertilisation ducts; Pl = parmula; S = spermathecae. Scale bars: 0.50 (**A–C**); 0.10 (**D–G**).

**Figure 12.**
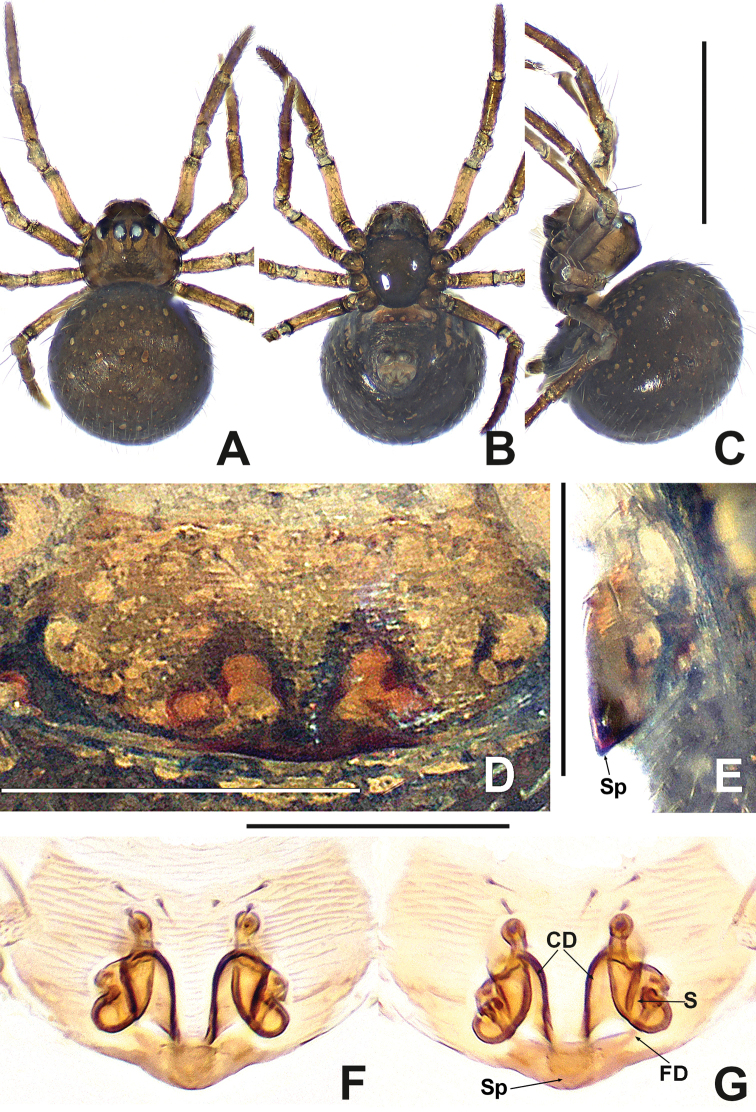
*Patuqiqi***A** female habitus, dorsal **B** female habitus, ventral **C** female habitus, lateral **D** epigyne, ventral **E** epigyne, lateral **F** vulva, ventral **G** vulva, dorsal. Abbreviations: CD = copulatory ducts; FD = fertilisation ducts; S = spermathecae; Sp = scape. Scale bars: 0.50 (**A–C**); 0.10 (**D–G**).

The male of *P.nigeri* differs from that of other congeners, except *P.dakou* sp. nov., *P.silho*, and *P.xiaoxiao*, by lacking an exposed embolus (fig. 4A and B in Lin and Li 2009), a median apophysis and a tegular process. The male differs from *P.dakou* sp. nov. by the smaller tegulum (~ ¾ size of that of *P.dakou* sp. nov.) (Fig. 3A vs. fig. 4A in Lin and Li 2009), from *P.silho* by the elongate oval palpal bulb (short oval in *P.silho*) (fig. 4A and B in Lin and Li 2009 vs. fig. 5A and B in Saaristo, 1996) and differs from *P.xiaoxiao* by the absence of a tegular process (finger-like tegular process in *P.xiaoxiao*) (fig. 4A and B in Lin and Li 2009 vs. Fig. 14A and B). The female of *P.nigeri* is similar to that of *P.putao* sp. nov. by the shape of the epigyne (Figs 10D, E, 11D and E) and to *P.qiqi* in the configuration of the vulva (Figs 10G and 12G), but it can be easily distinguished from *P.putao* sp. nov. by the short and straight copulatory ducts, rather than long and twisted as in *P.putao* sp. nov. (cf. Figs 10F–G and 11F–G) and from *P.qiqi* by the tongue-shaped parmula that does not obscure the copulatory openings, rather than an indistinct scape that hides the copulatory openings (cf. Figs 10G and 12G).

##### Description.

See Fig. 10A–G and Lin and Li (2009).

##### Distribution.

China (Yunnan) (Fig. 23).

#### Patu putao

Taxon classificationAnimaliaAraneaeSymphytognathidae

S. Li & Lin sp. nov.

C362EFE5-DB94-5132-8F92-12EF09F6C108

http://zoobank.org/3FE3C44A-F22B-4189-A2CC-3D5E4B48B47D

Figures 11 , 23

##### Type material.

***Holotype*** ♀ (IZCAS-Ar 41045) **Myanmar**: Kachin State, Putao, Hponkanrazi Wildlife Sanctuary, near Camp 3, (27.61352°N, 96.98333°E; 2691 m alt.), 11.V.2017, J. Wu and Z. Chen leg.

##### Etymology.

The specific epithet derives from the type locality; noun in apposition.

##### Diagnosis.

This new species is similar to *P.jiangzhou* sp. nov. and *P.nigeri* in the shape of the epigyne, the tongue-shaped parmula and the exposed copulatory openings, but it differs from *P.jiangzhou* sp. nov. by the rounded copulatory openings, the longer copulatory ducts and the larger, reniform spermathecae, rather than trumpet-shaped copulatory openings, shorter copulatory ducts and smaller, oval spermathecae (cf. Figs 11D and F–G vs. 5D and F–G) and from *P.nigeri* by the longer, twisted copulatory ducts that wrap around the spermathecae, rather than the shorter, straight copulatory ducts that do not wrap around the spermathecae (cf. Figs 11G and 10G).

##### Description.

**Female** (IZCAS-Ar 41045). Total length 0.68. Carapace 0.28 long, 0.28 wide, 0.28 high. Clypeus 0.10 high. Sternum 0.20 long, 0.20 wide. Abdomen 0.52 long, 0.56 wide, 0.56 high. Length of legs: I 0.76 (0.24, 0.08, 0.16, 0.12, 0.16); II 0.68 (0.20, 0.10, 0.12, 0.12, 0.14); III 0.60 (0.18, 0.08, 0.10, 0.12, 0.12); IV 0.74 (0.22, 0.10, 0.16, 0.12, 0.14).

**Somatic characters** (Fig. 11A–C). ***Colouration***: carapace centrally yellow, marginally pale brown. Chelicerae brown, endites and labium dark brownish. Sternum centrally yellow, brown at margins. Femora and patella brown-yellow, other segments dark brown. Abdomen light yellow, dark pigmentation around spinnerets and posterior. ***Prosoma***: carapace as long as wide, dorsally pear shaped. ALE > PME > PLE. PME contiguous, PER straight. Chelicerae anterior surface flat, densely covered with short setae. Endites rectangular. Labium subtriangular. Sternum plump, surface smooth, with sparse setae. ***Legs***: all patellae with 1 dorsal seta, tibia I and II with 2 dorsal setae, 1 on tibia III and IV. Metatarsi and tarsi densely covered with fine setae. ***Opisthosoma***: subrounded dorsally, postgaster area with short setae. Spinnerets dark.

***Epigyne*** (Fig. 11D–G): faintly sclerotised, internal structures faintly visible via the cuticle. Parmula tongue-shaped, protruded, bilateral basal corners concave. Copulatory openings round. Spermathecae kidney shaped, separated by ca. ½ – ⅓ their width. Copulatory ducts long, with a complex course, twisting around the spermathecae nearly 4 times. Fertilisation ducts short, starting at posterolateral margins of spermathecae.

**Male.** Unknown.

##### Distribution.

Myanmar (Fig. 23).

#### Patu qiqi

Taxon classificationAnimaliaAraneaeSymphytognathidae

Miller, Griswold & Yin, 2009

C9D976B1-50CD-5F7D-8354-7E2E41A9F771

Figures 12 , 23

Patu qiqi Miller, Griswold & Yin, 2009: 66, figs 65F–H, 67E, F, 73A and B (♂♀).

##### Type material.

***Holotype*** ♀ (CASENT 9029328, HNU) and ***paratypes*** 5♀, 2 juv. (CASENT 9029327, HNU) **China**: Yunnan Province, Gaoligongshan, Nujiang Prefecture, Nujiang State Nature Reserve, Qiqihe, 9.9 air km W of Gongshan (27.715°N, 98.565°E; 2000 m alt.), 9–14.VII.2000, H. Yan et al. leg.

##### Other material examined.

2♀ (NHMSU-HA122) **China**: Yunnan Province, Gongshan County, at 54 km of from Gongshan County to Dulongjiang Town, in primary forest, leaf litter (27.87840°N, 98.42274°E; 2525 m alt.), 13.VIII.2018, Y. Lin et al. leg.

##### Diagnosis.

The male differs from other *Patu* species, with the exception of *P.nigeri*, *P.silho* and *P.xiaoxiao*, by the palp with an unexposed embolus (fig. 73A and B in Miller et al. 2009). It differs by having a hooked median apophysis vs. lacking in *P.nigeri* and *P.silho* (cf. fig. 73A in Miller et al. 2009 and fig. 4A and B in Lin and Li 2009 and fig. 5A and B in Saaristo 1996) and it differs from *P.xiaoxiao* by lacking a tegular process (cf. fig. 73A in Miller et al. 2009 and Fig. 14A). The female is most similar to that of *P.nigeri* in the shape of the epigyne and the configuration of the vulva, but it can be easily distinguished by the indistinct scape and the hidden copulatory openings vs. a tongue-shaped parmula and exposed copulatory openings (cf. Figs 12F–G vs. 10F–G).

##### Description.

See Fig. 12A–G and Miller et al. (2009).

##### Distribution.

China (Yunnan) (Fig. 23).

#### Patu xiaoxiao

Taxon classificationAnimaliaAraneaeSymphytognathidae

Miller, Griswold & Yin, 2009

F957E33C-8191-5C62-BE12-5B46F8574DB2

**Figure 13.**
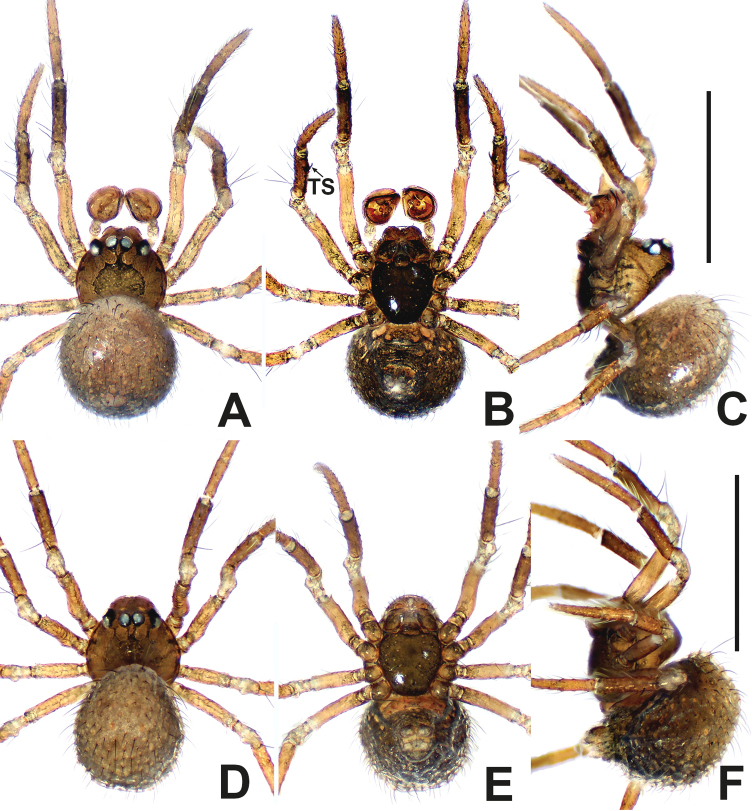
*Patuxiaoxiao***A** male habitus, dorsal **B** male habitus, ventral **C** male habitus, lateral **D** female habitus, dorsal **E** female habitus, ventral **F** female habitus, lateral. Abbreviation: TS = male clasping spines on tibia II. Scale bars: 0.50 (**A–F**).

**Figure 14.**
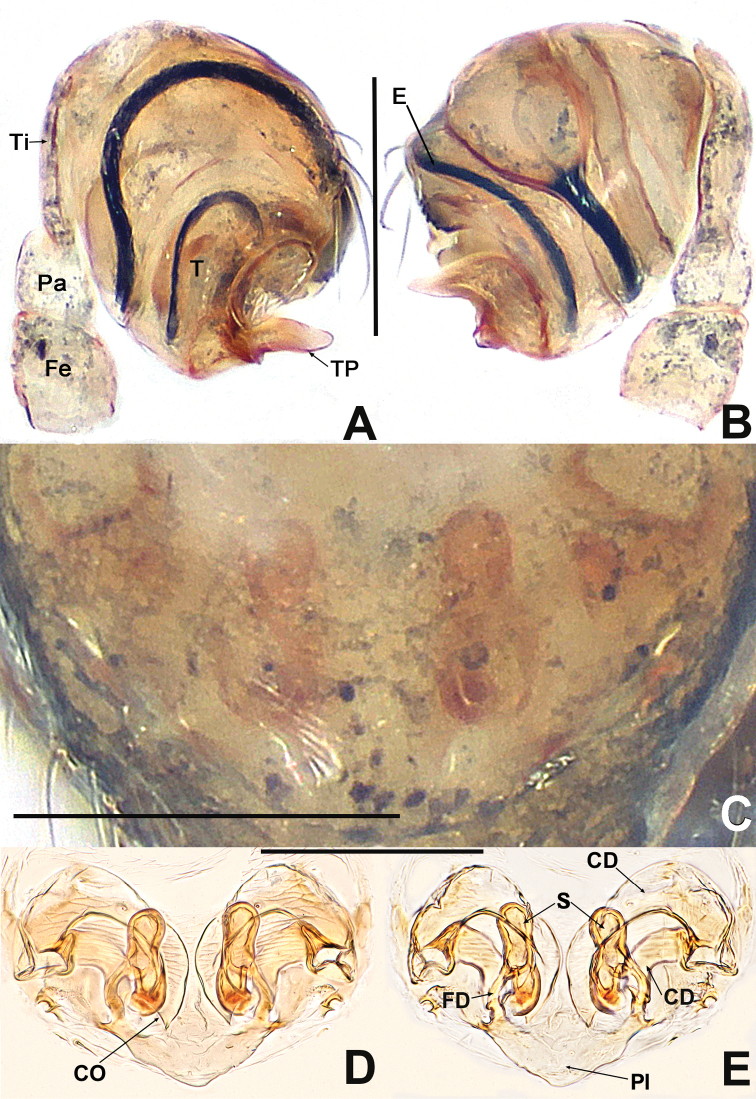
*Patuxiaoxiao***A** male palp, prolateral **B** male palp, retrolateral **C** epigyne, ventral **D** vulva, ventral **E** vulva, dorsal. Abbreviations: CD = copulatory ducts; Co = conductor; CO = copulatory opening; E = embolus; FD = fertilisation ducts; Fe = femur; MA = median apophysis; Pa = patella; Pl = parmula; S = spermathecae; T = tegulum; Ti = tibia; TP = tegular process. Scale bars: 0.10 (**A–E**).

Figures 13 , 14 , 23

Patu xiaoxiao Miller, Griswold & Yin, 2009: 67, fig. 67G and H (♀).

##### Type material.

***Holotype*** ♀ (CASENT 9022329, HNU) and ***paratypes*** 1♀ (CASENT 9029325, HNU) **China**: Yunnan Province, Lushui County, Pianma Township, Changyanhe River, 9.3 km of ESE Pianma, mixed broadleaf deciduous and evergreen forest, Winkler extraction of sifted leaf litter (25.99363°N, 98.66651°E; 2470 m alt.), 12.V.2005, C. Griswold leg.

##### Other material examined.

1♂ 1♀ (NHMSU-HA123) **China**: Yunnan Province, Lushui County, Pianma Township, Changyanhe River, 9.3 km of ESE Pianma, mixed broadleaf deciduous and evergreen forest, in leaf litter (25.99363°N, 98.66651°E; 2470 m alt.), 10.VIII.2018, Y. Lin et al. leg.; 1♂ (NHMSU-HA123) and 1♀ (NHMSU-HA123) used for sequencing, GenBank: MW970245 and MW970244, same data as preceding; 2♀ (NHMSU-HA124), Fugong County, Shiyueliang Town, along the road from Shiyueliang to Shibali Village, primary forest (27.15546°N, 98.80573°E; 2193 m alt.), 19.VIII.2018, Y. Lin et al. leg.

##### Diagnosis.

The male of *P.xiaoxiao* can be distinguished from other congeners, with the exception of *P.woodwardi*, by the stout bulb lacking a conductor or median apophysis and having a finger-like tegular process (Fig. 14A and B). It differs from *P.woodwardi* by having the entire embolus completely embedded in the bulb (cf. Fig. 14A and B and fig. 120 in Forster 1959). Females of *P.xiaoxiao* differs from those of other congeners by having a wide, triangular parmula, dumb-bell-shaped spermathecae separated by ~ 1.5× their width and arranged longitudinally in parallel, the copulatory ducts coiling into a loop and connecting to the postero-lateral corner of the spermathecae, the fertilisation ducts begin latero-medially on the spermathecae (Fig. 14C–E).

##### New morphological data.

**Male** (NHMSU-HA123). Total length 0.56. Carapace 0.28 long, 0.28 wide, 0.28 high. Clypeus 0.12 high. Sternum 0.20 long, 0.20 wide. Abdomen 0.36 long, 0.36 wide, 0.44 high. Length of legs: I 0.80 (0.20, 0.10, 0.20, 0.12, 0.18); II 0.72 (0.20, 0.10, 0.14, 0.12, 0.16); III 0.58 (0.14, 0.08, 0.14, 0.10, 0.12); IV 0.66 (0.18, 0.10, 0.14, 0.12, 0.12).

**Somatic characters** (Fig. 13A–C). ***Colouration***: carapace light brown, thoracic centre and margin with darker patches. Mouthparts nut brown, endites and labium black. Sternum black, with a few light, small dots. Leg colour light yellow gradually grading to very dark brown, tibia darkest brown. Abdomen dorsally light grey, laterally dark greyish, ventrally and posteriorly charcoal black. ***Prosoma***: carapace as long as wide, nearly round. Cephalic part vertical anteriorly and sloped posteriorly. Eyes, subequal in size. PER slightly recurved, ALE protruded. Chelicerae anterior surface flat. Labium semi-circular, wider than long. Sternum smooth, slightly plump. ***Legs***: each patella with 1 disto-dorsal seta, 1 proximal and 1 disto-dorsal seta on each tibia. Tibia II with 2 ventral clasping spines. ***Opisthosoma***: dorsally globose, laterally ovoid, clothed with black, long setae, cuticle rough with dots of varying shades and sizes. Spinnerets dark brown.

***Palp*** (Fig. 14A and B): size ~¼ of carapace. Femur swollen, wider than patella, patella as long as ~ ½ length of tibia. Tibia flat and lamellar. Tegulum smooth, with finger-like apical process. Embolus long, starting at retrolatero-medial part of tegulum, coiled into 2 loops inside bulb. Tip of embolus hidden within tegulum, not extended from top of bulb.

**Female.** Total length 0.56. Carapace 0.28 long, 0.28 wide, 0.24 high. Clypeus 0.08 high. Sternum 0.20 long, 0.20 wide. Abdomen 0.36 long, 0.32 wide, 0.40 high. Length of legs: I 0.70 (0.20, 0.06, 0.16, 0.14, 0.14); II 0.62 (0.14, 0.08, 0.14, 0.12, 0.14); III 0.52 (0.12, 0.06, 0.12, 0.08, 0.14); IV 0.60 (0.12, 0.08, 0.16, 0.10, 0.14).

**Somatic characters** (Fig. 13D–F). ***Colouration***: as in male, except lighter mouthparts and sternum. ***Prosoma***: carapace longer than wide, pear-shaped. Eye arrangement as in male. PER straight. Cephalic part lower than in male. ***Legs***: spination as in male, except for lack of clasping spines on tibia II. ***Opisthosoma***: subovoid in dorsal view, cuticle. Spinnerets dark grey.

***Epigyne*** (Fig. 14C–E): faintly sclerotised, internal structures nearly invisible via the cuticle. Parmula triangular, length equal to ca. ½ width, slightly protruded. Spermathecae nearly dumb-bell-shaped, longitudinally parallel, separated by ~ 1.5× their width. Copulatory openings large. Copulatory ducts translucent, their width equal to ca. 2.5× width of fertilisation ducts and folded at middle, distal part connected with postero-lateral part of spermathecae. Fertilisation duct shorter than a spermatheca length, narrow, originates from the lateral central position of spermathecae.

##### Distribution.

China (Yunnan) (Fig. 23).

##### Remarks.

*Patuxiaoxiao* was described, based on three females. Based on supplementary materials from the type locality collected in 2018, the male is described for the first time here.

#### Genus Kirinua

Taxon classificationAnimaliaAraneaeSymphytognathidae

S. Li & Lin gen. nov.

75D7B008-107C-5C1C-ADA6-8654F5823AD0

http://zoobank.org/5FD94CCF-CB91-485B-A2BF-6FABEDF07B53

##### Type species.

*Kirinuamaguai* sp. nov., from Guangxi, China.

##### Etymology.

The generic name is derived from Kirin, one of the most powerful creatures ever known in East Asia. The gender is masculine.

##### Diagnosis.

**Figure 15.**
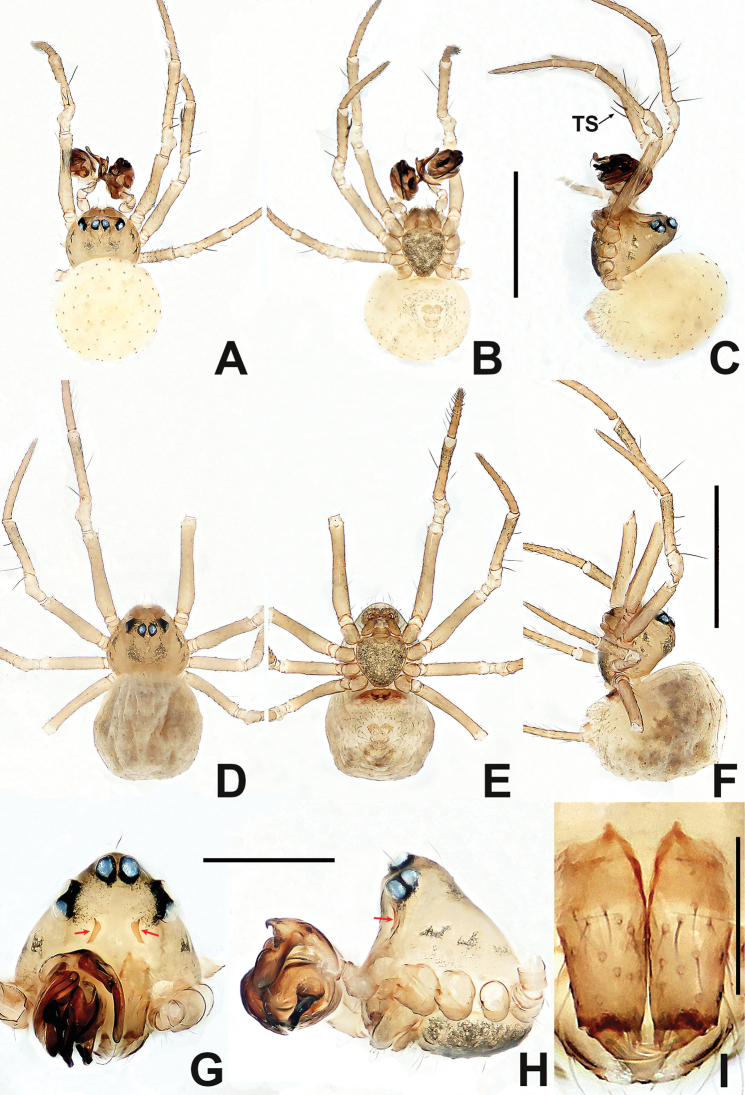
*Kirinuamaguai* sp. nov. **A** male habitus, dorsal **B** male habitus, ventral **C** male habitus, lateral **D** female habitus, dorsal **E** female habitus, ventral **F** female habitus, lateral **G** male prosoma, anterior **H** male prosoma, lateral **I** male chelicerae, anterior. Abbreviation: TS = male clasping spines on tibia II. Scale bars: 0.50 (**A–F**); 0.20 (**G, H**); 0.10 (**I**).

**Figure 16.**
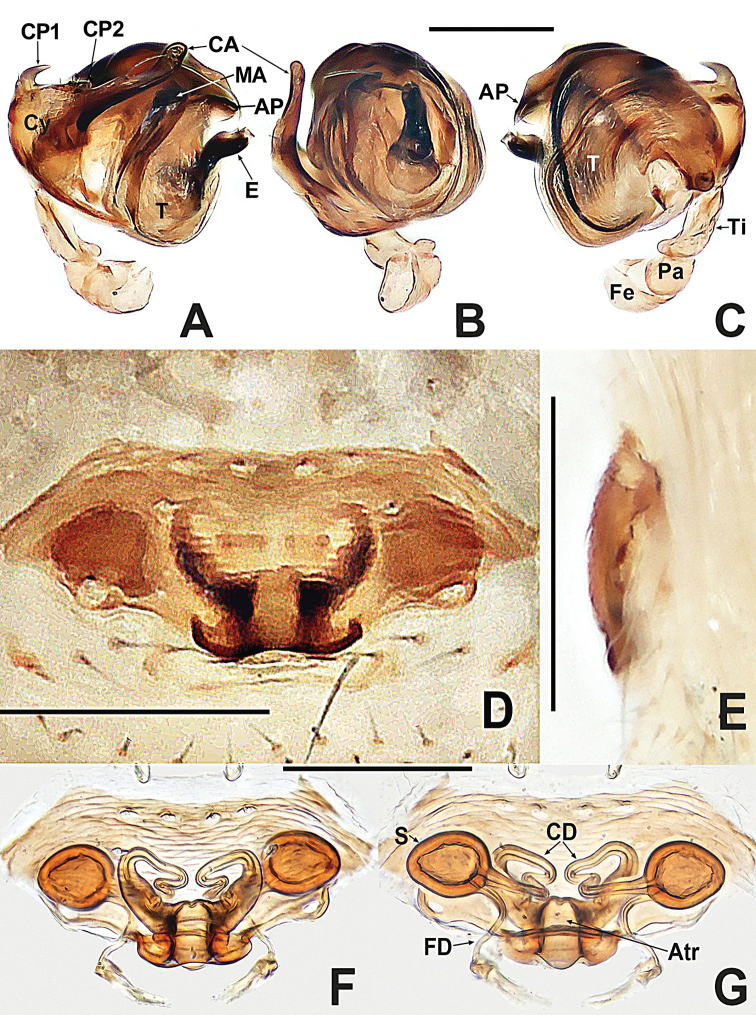
Kirinuamaguai sp. nov. **A** male palp, prolateral **B** male palp, ventral **C** male palp, retrolateral **D** epigyne, ventral **E** epigyne, lateral **F** vulva, ventral **G** vulva, dorsal. Abbreviations: Atr = atrium; AP = apical process; CA = cymbial apophysis; CD = copulatory ducts; Co = conductor; CO = copulatory opening; Cy = cymbium; CP1 = proximal cymbial process; CP2 = distal cymbial process; E = embolus; FD = fertilisation ducts; Fe = femur; MA = median apophysis; Pa = patella; S = spermathecae; T = tegulum; Ti = tibia. Scale bars: 0.10 (**A–G**).

**Figure 17.**
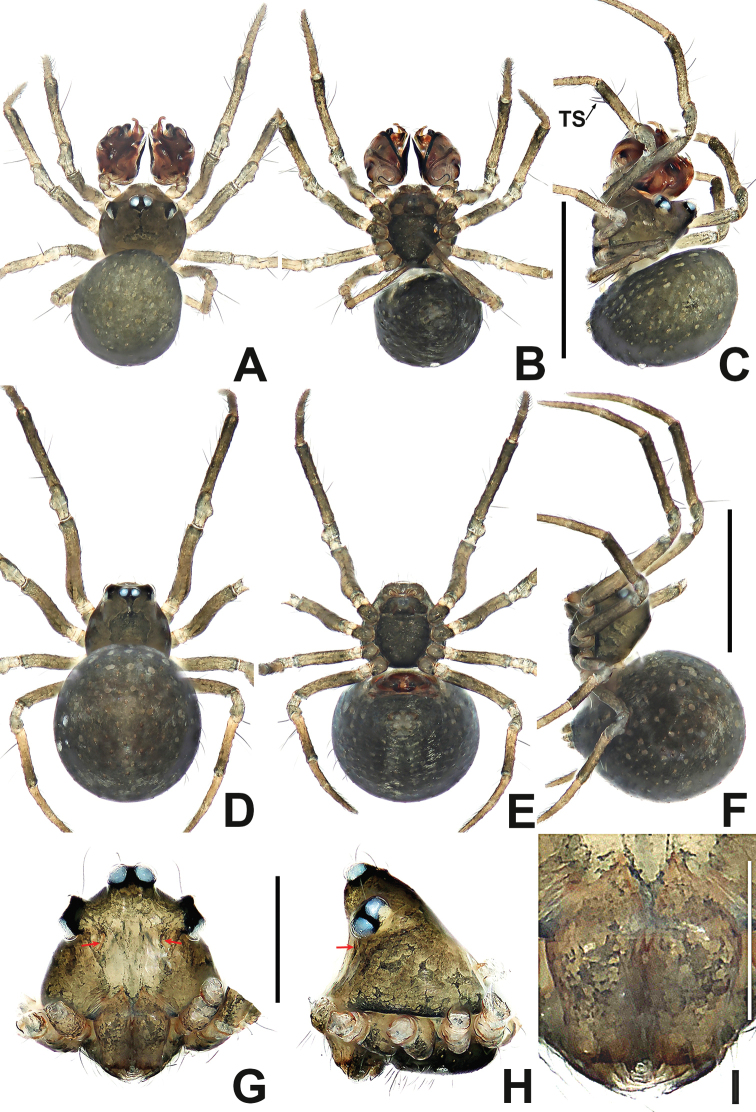
*Kirinuayangshuo* sp. nov. **A** male habitus, dorsal **B** male habitus, ventral **C** male habitus, lateral **D** female habitus, dorsal **E** female habitus, ventral **F** female habitus, lateral **G** male prosoma, anterior **H** male prosoma, lateral **I** male chelicerae, anterior. Abbreviation: TS = male clasping spines on tibia II. Scale bars: 0.50 (**A–F**); 0.20 (**G, H**); 0.10 (**I**).

**Figure 18.**
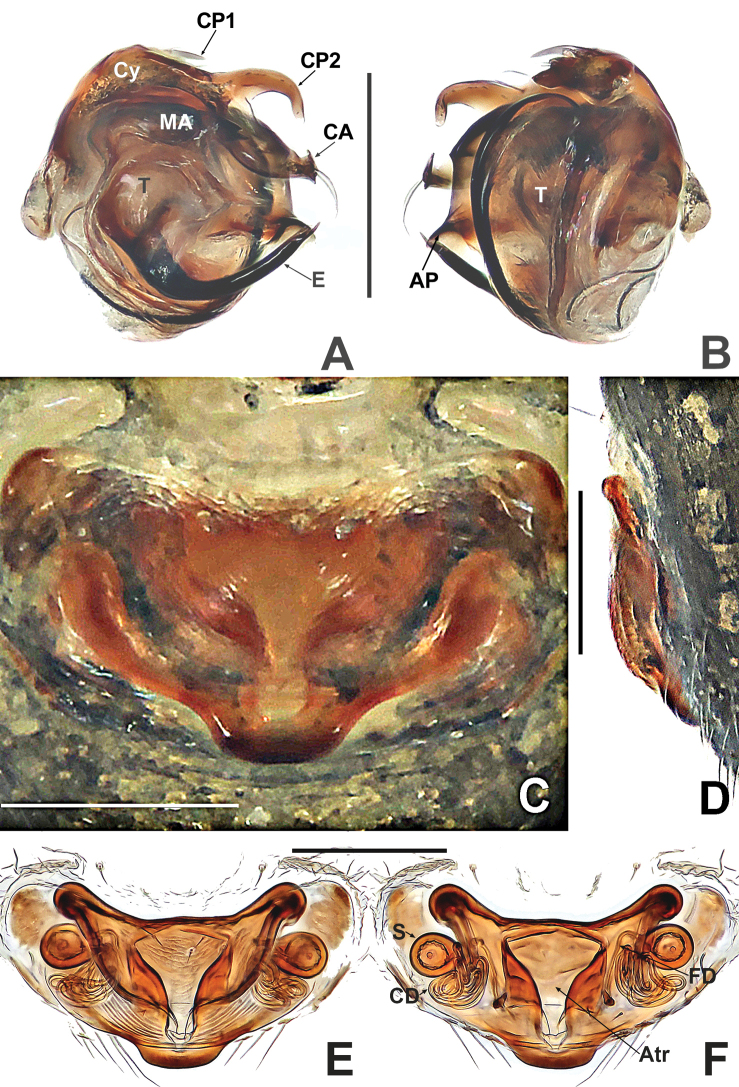
*Kirinuayangshuo* sp. nov. **A** male palp, prolateral **B** male palp, retrolateral **C** epigyne, ventral **D** epigyne, lateral **E** vulva, ventral **F** vulva, dorsal. Abbreviations: Atr = atrium; AP = apical process; CA = cymbial apophysis; CD = copulatory ducts; Co = conductor; CO = copulatory opening; Cy = cymbium; CP1 = proximal cymbial process; CP2 = distal cymbial process; E = embolus; FD = fertilisation ducts; MA = median apophysis; S = spermathecae; T = tegulum. Scale bars: 0.10 (**A–F**).

**Figure 19.**
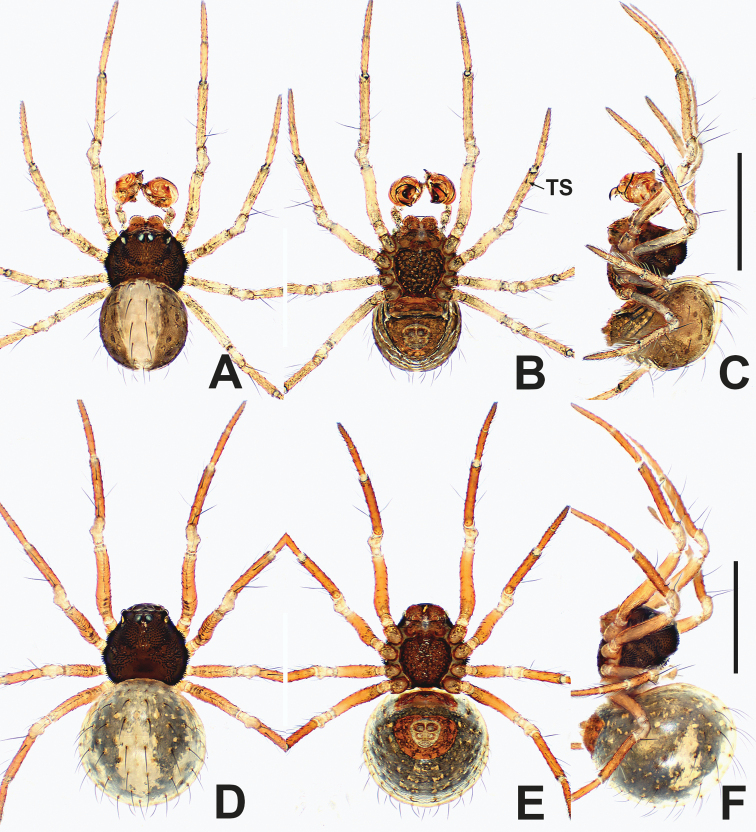
*Swildalongtou***A** male habitus, dorsal **B** male habitus, ventral **C** male habitus, lateral **D** female habitus, dorsal **E** female habitus, ventral **F** female habitus, lateral. Abbreviation: TS = male clasping spines on tibia II. Scale bars: 0.50 (**A–F**).

*Kirinua* gen. nov. can be distinguished from *Globignatha* and *Symphytognatha* by the chelicerae, which are fused only near the base (Figs 15I and 17I) vs. entirely fused (Balogh and Loksa 1968: fig. 10; Lin 2019: fig. 1H) and from *Anapistula* by having 6 eyes vs. 4 (except *A.boneti* Forster, 1958 with 6 eyes). It can be distinguished from *Anapogonia*, *Curimagua* and *Iardinis* by 6 eyes in three diads vs. 6 eyes in two triads and from *Crassignatha* and *Swilda* gen. nov. by lacking an abdominal scutum in males and a mostly smooth carapace in both sexes (Figs 15A–H and 17A–H) vs. abdominal scutum usually present in the male and carapace generally covered with tubercles or tiny thorn-like protrusions (Figs 19A, D, 21A and D; Li et al. 2020: fig. 1A–F; Rivera-Quiroz et al. 2021: figs 10b and 11b). *Kirinua* gen. nov. is similar to *Patu* in having 1–2 disto-ventral clasping spines on male tibia II, lacking an abdominal scutum latero-posteriorly in the male and the generally smooth carapace in both sexes (Figs 15A–H and 17A–H), but it can be distinguished by the male palp having cymbial structures (e.g. primary apophysis, process) and the female having nearly spherical spermathecae (Figs 16A, G, 18A and F) vs. male palp lacking cymbial structures and female with rod-shaped or oval spermathecae (Figs 3A, B, F, 9A, B and F).

##### Description.

Tiny, total length 0.60–0.80. Carapace rounded or pear-shaped dorsally, nearly triangular laterally (Figs 15A, 15D, 17A and 17D). Six eyes in 3 diads, cephalic part raised (Figs 15A, 15D, 17A and 17D). Clypeus concave, with pair of inverted, weakly sclerotised grooves in male (Figs 15G, H, 17G and H). Female lacking palps. Chelicerae fused at middle, with 2 adnate teeth (Figs 15I and 17I). Labium wider than long, fused to sternum, anterior margin with shallow notch in the middle (Figs 15B, E, 17B and E). Sternum heart-shaped, slightly plump, truncated posteriorly. Male tibia II with 2 subdistal ventral clasping spines (Figs 15C and 17C). Abdomen round in dorsal view, subovoid in lateral view, without posterior lobes or tubercles (Figs 15C, F, 17C and F). Anterior spinnerets larger than posteriors, median spinnerets hardly visible. Colulus absent.

***Male palp*** (Figs 16A–C, 18A and B): relatively large, ~ ½ size of carapace. Cymbium distinctly sclerotised, with 2 processes and a primary cymbial apophysis. Median apophysis present, finger-like, nearly as long as cymbial apophysis. Tegulum with a triangular apical process. Embolus short, stout, strongly sclerotised, tip furcate or blunt.

***Epigyne*** (Figs 16D–G and 18C–F): distinctly sclerotised. Scape present, inconspicuous, inflexible. Spermathecae globose, separated by at least 2 diameters. Copulatory ducts long, proximally fused, expanded into a broad atrium, distally curved or coiled between spermathecae. Fertilisation ducts short, thin. Inlet of copulatory duct and outlet of fertilisation duct nearly located at same position on spermatheca.

##### Composition.

*Kirinuamaguai* sp. nov. and *K.yangshuo* sp. nov.

##### Distribution.

China (Guangxi) (Fig. 23).

##### Relationships.

*Kirinua* gen. nov. is characterised by their tiny size, chelicerae fused at mid-line, AMEs and book lungs absent, female lacking palps and tarsi much longer than metatarsi. This new genus is similar to *Patu* by having 2 clasping spines on male tibia II, lacking an abdominal scutum latero-posteriorly in the male and the carapace of both sexes lacks modified pits or sculpturing (Figs 15A–H and 17A–H). The new genus differs from *Patu* by the highly modified structures of the cymbium (e.g. primary apophysis, process) and the epigyne has nearly spherical spermathecae and a broad atrium (Figs 16A, G, 18A and F).

#### Kirinua maguai

Taxon classificationAnimaliaAraneaeSymphytognathidae

S. Li & Lin sp. nov.

12C1B673-A9A9-5A2C-97BD-D45B33F930CF

http://zoobank.org/F32044F2-1449-4A5F-BC7F-5EC66AB5596A

Figures 15 , 16 , 23

##### Type material.

***Holotype*** ♂ (IZCAS-Ar 41046) and ***paratype*** 1♀ (IZCAS-Ar 41047) **China**: Guangxi Zhuang Autonomous Region, Hechi City, Fengshan County, Pingle Township, Maguai Cave (24.43194°N, 106.96737°E, 618 m alt.), 23.III.2015, Y. Li and Z. Chen leg.; 1♀ (NHMSU-HA008) used for sequencing, GenBank: MW970250, same data as for preceding.

##### Other material examined.

1♀ (NHMSU-HA005) **China**: Guangxi Zhuang Autonomous Region, Hechi City, Fengshan County, Pingle Township, Sanmen Cave (24.43163°N, 106.97124°E, 659 m alt.), 23.III.2015, Y. Li and Z. Chen leg.; 1♀ (NHMSU-HA011) same Province and County, Fengcheng Township, nameless cave (24.31023°N, 107.00213°E, 402 m alt.), 24.III.2015, Y. Li and Z. Chen leg.; 1♂ prosoma (NHMSU-HA016) same region, Hechi City, Donglan County, Bala Township, nameless cave (24.44368°N, 107.34726°E, 385 m alt.), 18.III.2015, Y. Li and Z. Chen leg.

##### Etymology.

The specific epithet derives from the name of the type locality; noun in apposition.

##### Diagnosis.

Males of the new species can be distinguished from those of *K.yangshuo* sp. nov. by the shorter, distally blunt embolus vs. a distally sharp, longer embolus (ca. 2× length of the former) and by a blunt cymbial apophysis vs. a truncated cymbial apophysis (Figs 16A and 18A). The female differs from that of *K.yangshuo* sp. nov. by the small atrium without a knob-shaped lateral hump vs. a large atrium with a knob-shaped lateral hump and by the shorter, copulatory duct coiled less than 2 times vs. the longer copulatory duct coiled more than 5 times (Figs 16F–G and 18E–F).

##### Description.

**Male** (IZCAS-Ar 41046). Total length 0.64. Carapace 0.32 long, 0.28 wide, 0.32 high. Clypeus 0.10 high. Sternum 0.20 long, 0.16 wide. Abdomen 0.44 long, 0.44 wide, 0.52 high. Length of legs: I 1.08 (0.30, 0.12, 0.24, 0.18, 0.24); II 0.98 (0.30, 0.12, 0.20, 0.14, 0.22); III 0.74 (0.20, 0.10, 0.14, 0.16, 0.14); IV 0.94 (0.32, 0.10, 0.20, 0.12, 0.20).

**Somatic characters** (Fig.. 15A–C and G–I). ***Colouration***: carapace pale yellow, with irregular darker patches at thoracic area and margins. Mouthparts pale brown. Sternum light grey. Legs pale yellow. Abdomen pale. ***Prosoma***: carapace longer than wide, as long as high. ALE largest, PME smallest, PER slightly recurved. Clypeus slightly concave. Clypeal notches separated by width of PME (Fig. 15G). Chelicerae covered with sparse, long setae anteriorly. Endites longer than wide. Labium wider than long, with shallow notch on anterior margin. Sternum slightly plump. ***Legs***: each patella with 1 disto-dorsal seta, tibia with 2 dorsal setae, 1 subproximal and 1 subdistal, metatarsus I with 1 subproximal dorsal seta. ***Opisthosoma***: round in dorsal view and ovoid in lateral view, with sparse, long setae, posteriorly extended beyond spinnerets. Spinnerets light yellow.

***Palp*** (Fig.. 16A–C): strongly sclerotised. Femur and patella swollen, tibia longer than femur or patella, with a small retrolateral basal tubercle. Cymbium large, with 1 hook-like process, 1 nodular process with few short setae and 1 long, finger-like distal cymbial apophysis. Bulb flattened. Median apophysis strip-shaped, located below cymbial apophysis. Rugose tegulum with triangular apical process. Embolus stiff, shorter than median apophysis, slightly bent at middle, blunt distally.

**Female** (IZCAS-Ar 41047). Total length 0.64. Carapace 0.32 long, 0.32 wide, 0.28 high. Clypeus 0.10 high. Sternum 0.20 long, 0.20 wide. Abdomen 0.44 long, 0.40 wide, 0.48 high. Length of legs: I 1.10 (0.34, 0.14, 0.22, 0.18, 0.22); II 0.96 (0.28, 0.12, 0.20, 0.16, 0.20); III 0.78 (0.24, 0.10, 0.12, 0.14, 0.18); IV 0.92 (0.26, 0.12, 0.20, 0.14, 0.20).

**Somatic characters** (Fig.. 15D–F). ***Colouration***: same as in male. ***Prosoma***: carapace nearly pear-shaped in dorsal view. Cephalic part elevated, lower than in male. PER slightly procurved. ***Legs***: spination of each leg as in male. ***Opisthosoma***: as in male, except for wrinkled abdominal cuticle that may be caused by ethanol dehydration.

***Epigyne*** (Fig.. 16D–G) internal structures faintly visible via the translucent epigynal cuticle. Scape barely visible. Vulva relatively complex. Spermathecae subglobose, close to posterior margin, separated by about 2 diameters. Fertilisation duct thinner than copulatory duct, located dorso-posteriorly on copulatory duct, originates from posteromedial margin of spermatheca, curved outwards at sides of atrium, then extended downwards (Fig. 16G).

##### Distribution.

China (Guangxi) (Fig. 23).

#### Kirinua yangshuo

Taxon classificationAnimaliaAraneaeSymphytognathidae

S. Li & Lin sp. nov.

B1D1BECD-645A-53E7-A7C8-EED9E9CF18B6

A5AFCA25-600D-4825-ACA6-91733DFC91B8

Figures 17 , 18 , 23

##### Type material.

***Holotype*** ♂ (IZCAS-Ar 41048) and ***paratypes*** 2♀ (IZCAS-Ar 41049, 41050) **China**: Guangxi Zhuang Autonomous Region, Guilin City, Yangshuo County, Xinping Township, Bingshiyan Cave (24.94477°N, 110.60615°E), 11.I.2013, J. Du and X. Wang leg.; 1♂ juvenile (NHMSU-HA018) and 1♀ (NHMSU-HA018) used for sequencing, GenBank: MW970236 and MW970235, same data as for preceding.

##### Etymology.

The specific epithet derives from the name of the type locality; noun in apposition.

##### Diagnosis.

See diagnosis for *K.maguai* sp. nov.

##### Description.

**Male** (IZCAS-Ar 41048). Total length 0.60. Carapace 0.28 long, 0.24 wide, 0.36 high. Clypeus 0.20 high. Sternum 0.16 long, 0.16 wide. Abdomen 0.28 long, 0.28 wide, 0.48 high. Length of legs: I 1.04 (0.30, 0.12, 0.24, 0.14, 0.24); II 0.86 (0.24, 0.12, 0.20, 0.10, 0.20); III 0.66 (0.16, 0.10, 0.12, 0.10, 0.18); IV 0.82 (0.26, 0.10, 0.16, 0.14, 0.16).

**Somatic characters** (Fig. 17A–C and G–I). ***Colouration***: body dark, nearly black. Legs light brown, with black pigmentation. ***Prosoma***: carapace longer and higher than wide. Cephalic apex at PME position. ALE > PLE = PME, PME separated by ca. half a radius, PER recurved. Clypeus concave, paired notches separated by more than width of PME (Fig. 17G and H). Labium short, with shallow notch. ***Legs***: each patella with 1 long dorsal seta, each tibia with 2 long dorsal setae. ***Opisthosoma***: shape as in *K.maiguai* sp. nov., spinnerets dark.

***Palp*** (Fig. 18A and B): strongly sclerotised. Proximal cymbial process (CP1) small, sharp, needle-like, distal cymbial process (CP2) large, hooked. Cymbial apophysis (CP) truncated, with 2 distal long setae. Distal bifurcation of median apophysis located directly below the hooked CP2 (Fig. 18A). Tegulum translucent, weakly rugose, with a triangular apical process. Embolus longer than median apophysis, robust, horn-like, strongly sclerotised, gradually tapering, bent at nearly proximal ⅓, forked at terminus.

**Female** (IZCAS-Ar 41049). Total length 0.80. Carapace 0.32 long, 0.28 wide, 0.32 high. Clypeus 0.10 high. Sternum 0.20 long, 0.20 wide. Abdomen 0.52 long, 0.52 wide, 0.60 high. Length of legs: I 1.20 (0.40, 0.10, 0.30, 0.18, 0.22); II 0.90 (0.22, 0.12, 0.22, 0.14, 0.20); III 0.74 (0.20, 0.10, 0.14, 0.10, 0.20); IV 0.86 (0.26, 0.10, 0.18, 0.12, 0.20).

**Somatic characters** (Fig. 17D–F). ***Colouration***: same as in male. ***Prosoma***: carapace longer than wide, as long as high. Cephalic part lower than in male, flat dorsally. PER slightly recurved. ***Legs***: spination of each leg as in male. ***Opisthosoma***: laterally oviform. Spinnerets Located ventrally.

***Epigyne*** (Fig. 18C–F): plate wider than long, strongly sclerotised. Scape wider than long, slightly protruded. Spermathecae globose, separated by more than 3.5 diameters. Copulatory ducts long, their proximal parts enlarged, forming a broad, inverted, subtriangular atrium, with knob-shaped lateral humps; middle part coiled more than 5 times; distal part connected longitudinally to spermatheca. Fertilisation duct slightly bent, runs along lateral wall of atrium, originating above coiled part of copulatory duct.

##### Distribution.

China (Guangxi) (Fig. 23).

#### Genus Swilda

Taxon classificationAnimaliaAraneaeSymphytognathidae

S. Li & Lin gen. nov.

7FABCB5C-4AC4-5FE3-B620-01B09356C397

http://zoobank.org/CC843E39-93C5-44A5-9C50-5E7ABBF17B21

##### Type species.

*Crassignathalongtou* Miller, Griswold & Yin, 2009, from Gaoligong Mountain, south-western China.

##### Etymology.

The generic name *Swilda* is derived from the Swild Studio (in Chinese: Xi Nan Shan Di Gong Zuo Shi). It is named after the organisation in honour of its dedication to promoting public advocacy for wildlife conservation and nature education in southwest China. The gender is masculine.

##### Diagnosis.

**Figure 20.**
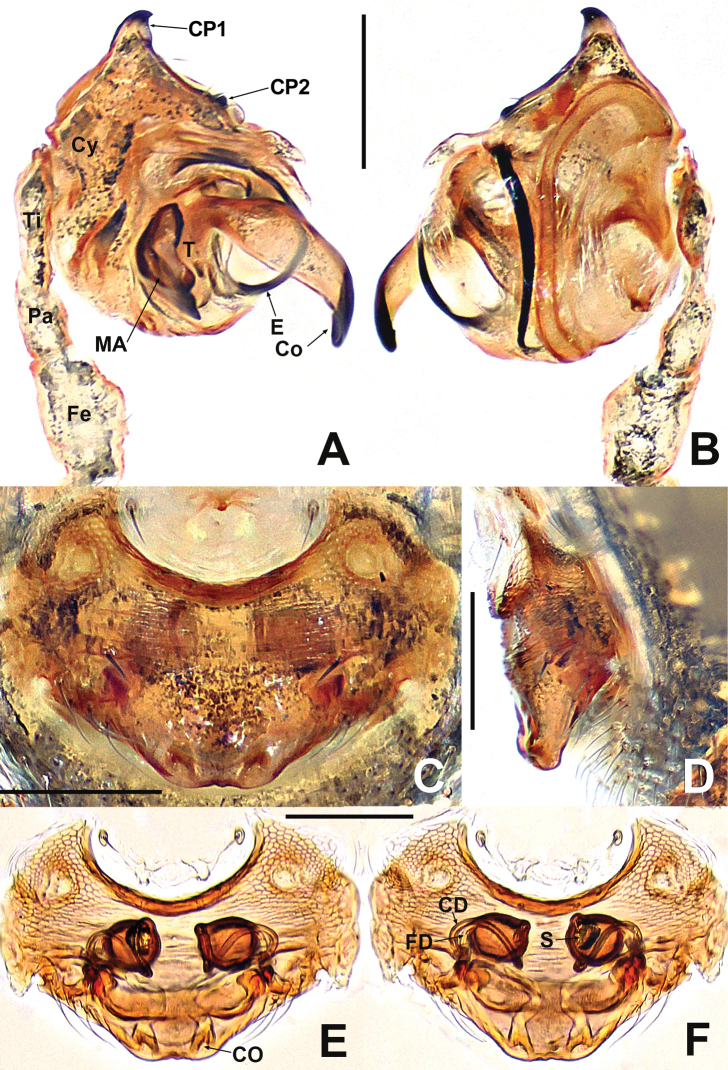
*Swildalongtou***A** male palp, prolateral **B** male palp, retrolateral **C** epigyne, ventral **D** epigyne, lateral **E** vulva, ventral **F** vulva, dorsal. Abbreviations: CA = cymbial apophysis; CD = copulatory ducts; Co = conductor; CO = copulatory opening; Cy = cymbium; CP1 = proximal cymbial process; CP2 = distal cymbial process; E = embolus; FD = fertilisation ducts; Fe = femur; MA = median apophysis; Pa = patella; S = spermathecae; T = tegulum; Ti = tibia. Scale bars: 0.10 (**A–F**).

**Figure 21.**
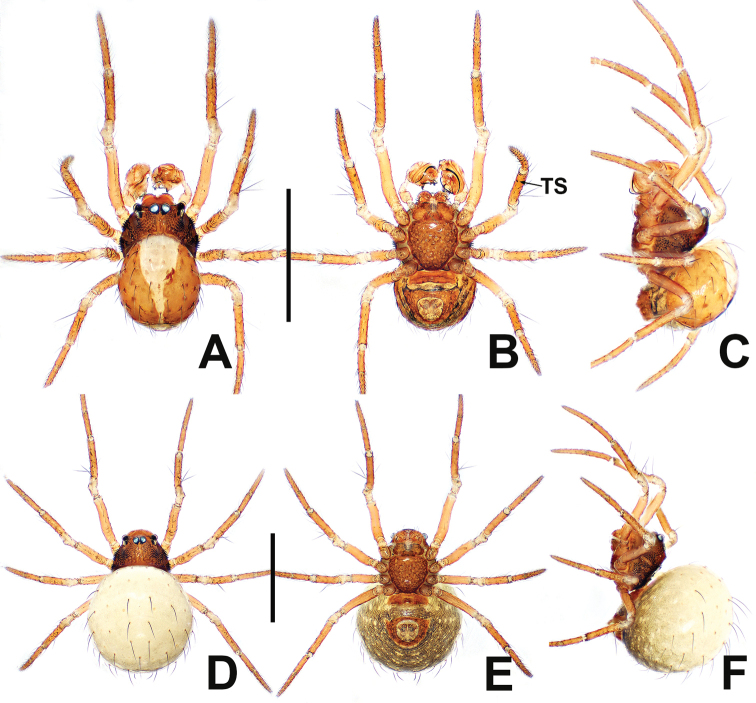
*Swildaspinathoraxi***A** male habitus, dorsal **B** male habitus, ventral **C** male habitus, lateral **D** female habitus, dorsal **E** female habitus, ventral **F** female habitus, lateral. Abbreviation: TS = male clasping spines on tibia II. Scale bars: 0.50 (**A–F**).

**Figure 22.**
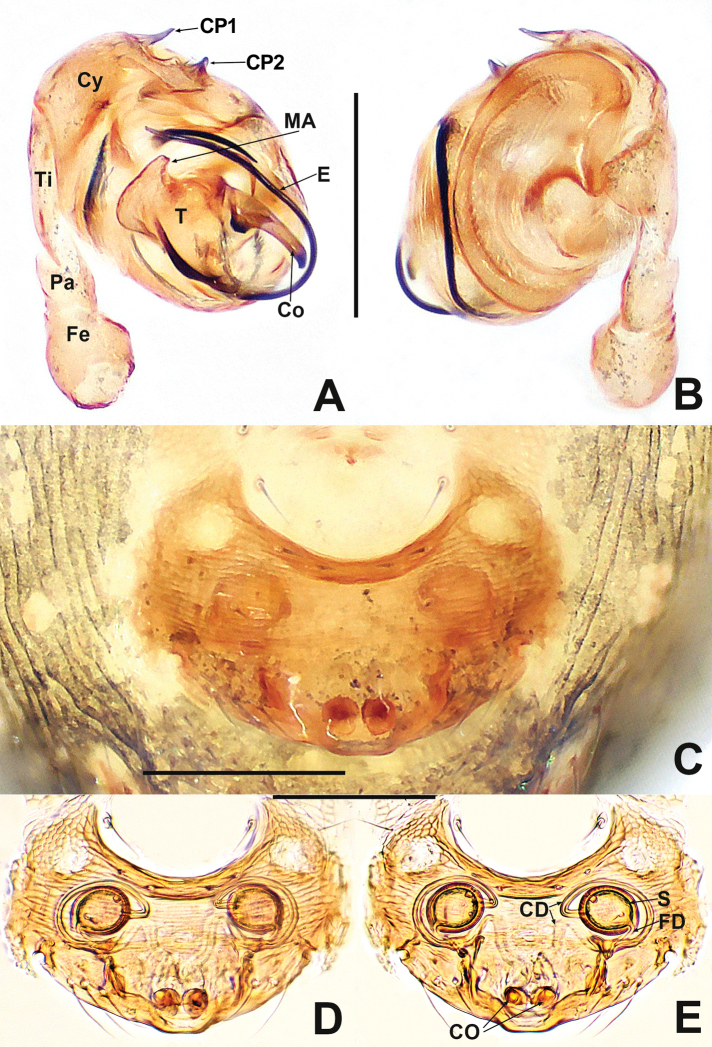
*Swildaspinathoraxi***A** male palp, prolateral **B** male palp, retrolateral **C** epigyne, ventral **D** vulva, ventral **E** vulva, dorsal. Abbreviations: CA = cymbial apophysis; CD = copulatory ducts; Co = conductor; CO = copulatory opening; Cy = cymbium; CP1 = proximal cymbial process; CP2 = distal cymbial process; E = embolus; FD = fertilisation ducts; Fe = femur; MA = median apophysis; Pa = patella; S = spermathecae; T = tegulum; Ti = tibia. Scale bars: 0.10 (**A–E**).

*Swilda* gen. nov. is easily distinguished from other symphytognathids, except *Crassignatha*, by having an anteromedially-split dorsal scutum in the male and a highly ornamented spinous and pitted carapace in both sexes (Figs 19A, D, 21A and D). It resembles *Crassignatha* in carapace texture and the spherical spermathecae. The male differs from those of *Crassignatha* by having a conductor and lacking a cymbial tooth (Figs 20A and 22A) vs. lacking a conductor, but having a cymbial tooth (figs 2A, 8A and 10A in Li et al. 2020); the female differs in lacking a scape and by the separated copulatory openings (Figs 20F and 22E) vs. having a protruded scape and the adnate copulatory openings located at the apex of the scape in *Crassignatha* (figs 2G, 4G and 8G in Li et al. 2020).

##### Description.

Minute, body length 0.50–1.00. Carapace rounded or pyriform, strongly sclerotised, surface spinous and pitted (Figs 19A, 19D, 21A and 21D). Cephalic part raised, higher in male than in female. Six eyes, white, in 3 diads. Clypeus high, more than 2× diameter of ALE, concave. Chelicerae fused at middle, with 1 bifid tooth. Labium tongue-shaped, fused to coarse, pitted sternum. Sternum heart-shaped, slightly plump, truncated posteriorly (Figs 19B, 19E, 21B and 21E). Male tibia II with one clasping spine (Figs 19B and 21B). Abdomen globose in both sexes, male usually with a weakly sclerotised abdominal scutum split at mid-line (Figs 19A and 21A), sclerotised annular plate encircles spinnerets (Figs 19B, 19E, 21B and 21E). Colulus absent.

Pedicel orifice wide, wider than epigyne, with 2 pairs of lateral setae, posterior margin rebordered. Epigastric scutum distinctly sclerotised ventrally (not encircling pedicel).

***Male palp*** (Figs 20A, B, 22A and B): femur swollen, wider than patella, tibia lamellar. Bulb oblate; cymbium well developed, covers bulb on prolatero-ventral side, with 2 processes (CP1, CP2). Median apophysis present. Conductor longer than median apophysis, protruded out of bulb. Embolus long, tubular, sclerotised, originates at prolateral margin of tegulum, curved and extended beneath distal part of cymbium.

***Epigyne*** (Figs 20C–F and 22C–E): sclerotised, posterior margin slightly protruded. Parmula inconspicuous. Copulatory openings separated, located at posterior margin. Spermathecae globose, separated by less than 2 diameters. Copulatory ducts slender, twisted, encircling spermathecae, connected to anteromedial surface of spermathecae. Fertilisation ducts originate at posterolateral surface of spermathecae.

##### Composition.

*Swildalongtou* (Miller et al, 2009) comb. nov. and *S.spinathoraxi* (Lin & Li, 2009) comb. nov.

##### Relationships.

*Swilda* gen. nov. is characterised by its tiny size, fused chelicerae at mid-line, AMEs and book lungs absent, female lacking palps and tarsi much longer than metatarsi. Here, the male of *C.longtou* is described for the first time and specimens of *P.spinathoraxi* are re-examined. We found the morphological features of these two species to be very similar to those of *Crassignatha* (see Li et al. 2020: 65), sharing the following combination of characters: a clasping spine on tibia II and an abdominal scutum latero-posteriorly in the male and a decorated carapace and sclerotised epigastric scutum in both sexes (Figs 19A–F and 21A–F). The differences between these two species and *Crassignatha* are: a pitted and spinous carapace, a sclerotised annular plate that encircles the spinnerets (cf. Figs 19A–F and 21A–F vs. figs 1A–F and 7A–F in Li et al. 2020), only 1 male clasping spine (cf. Figs 19B and 21B vs. figs 1B and 12C in Li et al. 2020: only 1 spine in a few species), male palps lack a cymbial tooth, but have a conductor (cf. Figs 20A and 22A vs. figs 2A and 8A in Li et al. 2020) and the epigyne lacks a protruded scape (cf. Figs 20E–F and 22E–E vs. figs 2E and 6E in Li et al. 2020).

The genetic distance we estimated, based on COI, also indicated differences between these two species and members of other genera (see Appendix Table A1). Phylogenetic analysis of molecular data indicates that *P.spinathoraxi* and *C.longtou* are clearly congeneric. Additionally, the combined genetic evidence from five genes supports the monophyly of *Swilda* gen. nov. and the sister group relationship of the two genera (unpubl. data). Therefore, *Swilda* gen. nov. is proposed as a new genus in which to place *S.longtou* (Miller et al, 2009) comb. nov., transferred from *Crassignatha* and *S.spinathoraxi* comb. nov., transferred from *Patu*. We designate *Swildalongtou* as the type species for this new genus.

##### Distribution.

**Figure 23.**
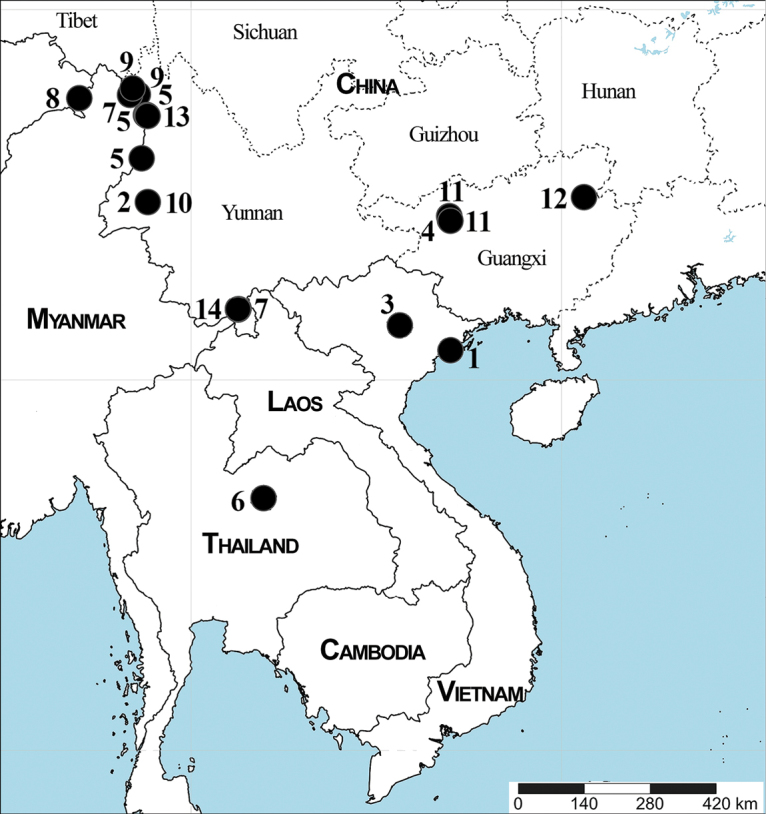
Distribution records of fourteen species of genera *Patu*, *Kirinua* gen. nov. and *Swilda* gen. nov. in Asia **1***P.catba* sp. nov. **2***P.dakou* sp. nov. **3***P.damtao* sp. nov. **4***P.jiangzhou* sp. nov. **5***P.jidanweishi***6***P.nagarat* sp. nov. **7***P.nigeri***8***P.putao* sp. nov. **9***P.qiqi***10***P.xiaoxiao***11***K.maguai* sp. nov. **12***K.yangshuo* sp. nov. **13***S.longtou***14***S.spinathoraxi.*

China (Yunnan) (Fig. 23).

#### Swilda longtou

Taxon classificationAnimaliaAraneaeSymphytognathidae

, (Miller et al., 2009) comb. nov.

E0140CAF-0FC6-5BB9-AE05-A1CFA24E89BE

Figures 19 , 20 , 23

Crassignatha longtou Miller, Griswold & Yin, 2009: 76, figs 89E, F, 90A–C, 91A–F and 92A–D (♀).

##### Type material.

***Holotype*** ♀ (CASENT 9029292, HNU) and ***paratypes*** 3♀, 1 juv. (CASENT 9020733, HNU), 2♀ (CASENT 9020732, HNU) **China**: Yunnan Province, 10 km of W Nujiang on Shibali Rd., N fork, Yamu He, Gaoligongshan, moist earthen embankments (27.13795°N, 98.82240°E; 1850 m alt.), 25.IV.2004, C. Griswold leg.; 1♀ (CASENT 9020740, HNU): Yunnan Province, Fugong County, 4.5 km N of Aludi Village, 22.1 km N of Fugong, in stream gorge (26.10829°N, 98.87162°E; 1250 m alt.), 23.IV.2004, C. Griswold leg.

##### Other material examined.

5♂, 10♀ (NHMSU-HA112) **China**: Yunnan Province, 10 km of W of Nujiang on Shibali Rd., N fork, Yamu He, Gaoligongshan, moist earthen embankments (27.13795°N, 98.82240°E; 1850 m alt.), 19.VIII.2018, Y. Lin et al. leg.; 1♂ (NHMSU-HA112) and 1♀ (NHMSU-HA112) used for sequencing, GenBank: MW970249 & MW970241, same data as for preceding. 5♂, 11♀ (NHMSU-HA111): Yunnan Province, Fugong County, Shilajia Village, Yamu He (27.13440°N, 98.82625°E; 1792 m alt.), 19.VIII.2018, Y. Lin et al. leg.

##### Diagnosis.

The male of *S.longtou* can be distinguished from that of *S.spinathoraxi* by the larger proximal cymbial process (CP1), the human-ear-shaped median apophysis and the wider and longer conductor (Fig. 20A) vs. needle-like proximal cymbial process (CP1), mastoid median apophysis and narrower and shorter conductor (Fig. 21A). The female differs by the separated copulatory openings, spermathecae separated by less than one diameter in *S.longtou* vs. adjacent copulatory openings, spermathecae separated by more than one diameter in *S.spinathoraxi* (cf. Figs 20E and F and 22D and E).

**Male** (NHMSU-HA112). Total length 0.68. Carapace 0.32 long, 0.36 wide, 0.36 high. Clypeus 0.16 high. Sternum 0.24 long, 0.24 wide. Abdomen 0.44 long, 0.44 wide, 0.48 high. Length of legs: I 1.24 (0.38, 0.14, 0.30, 0.20, 0.22); II 1.00 (0.30, 0.12, 0.22, 0.16, 0.20); III 0.80 (0.20, 0.10, 0.18, 0.14, 0.18); IV 0.96 (0.26, 0.12, 0.24, 0.18, 0.16).

**Somatic characters** (Fig. 19A–C). ***Colouration***: carapace and sternum dark brown. Chelicerae, endites and labium brown. Abdomen pale dorsally, fuscous laterally and ventrally. ***Prosoma***: PME separated by ~ ¾ their diameter. ALE protruded, PER slightly recurved. Cervical groove distinct, thoracic fovea shallow. Sternum slightly plump, surface coarse with pits, truncated posteriorly. ***Legs***: light brown, distal tibia darker, femora I and II slightly swollen basally. Patella with 1 long dorso-distal seta. Tibia I and II with 2 long dorsal setae, 1 on tibia III and IV. Tibia II with 1 large subdisto-ventral spine. ***Opisthosoma***: spherical in dorsal view, with sparse, long setae, with a posterolateral scutum. Spinnerets brown, surrounded by a circular plate.

***Palp*** (Fig. 20A and B): bulb oblate, ~ 1/3 size of carapace. Cymbium broad retrolaterally, with 2 sclerotised processes, large proximally (CP1) and small distally (CP2). Tegulum smooth. Median apophysis human-ear-shaped. Conductor large, longer than wide, basally constricted, distally curved. Sperm duct originates at prolateral base of bulb, embedded in the bulb. Embolus long, tubular, strongly sclerotised, mesally curved and distally extended below apex of cymbium.

**Female** (NHMSU-HA112). Total length 0.92. Carapace 0.36 long, 0.36 wide, 0.32 high. Clypeus 0.16 high. Sternum 0.24 long, 0.24 wide. Abdomen 0.56 long, 0.56 wide, 0.64 high. Length of legs: I 1.10 (0.28, 0.14, 0.30, 0.18, 0.20); II 0.96 (0.22, 0.14, 0.22, 0.16, 0.22); III 0.88 (0.22, 0.12, 0.20, 0.12, 0.22); IV 0.94 (0.28, 0.10, 0.22, 0.14, 0.20).

**Somatic characters** (Fig. 19D–F). Habitus features and modifications as in male, but without postero-lateral scutum.

***Epigyne*** (Fig. 20C–F): sclerotised, with 2 macrosetae and some setae (Fig. 20C). Internal structures faintly visible via translucent cuticle. Globular spermathecae separated by slightly less than one diameter. Fertilisation ducts short, originating anterolaterally on spermathecae. Copulatory ducts long, arising postero-laterally on spermathecae, coiling 1¼ times around spermathecae from copulatory openings.

##### Distribution.

China (Yunnan) (Fig. 23).

#### Swilda spinathoraxi

Taxon classificationAnimaliaAraneaeSymphytognathidae

, (Lin & Li, 2009) comb. nov.

6909B43A-889A-5A77-AC06-C57DF2AE59E6

Figures 21 , 22 , 23

Patu spinathoraxi Lin & Li, 2009: 60, figs 14A, B, 15A, B, 16A–E, 17A and B (♂♀).

##### Type material.

***Holotype*** ♀ (IZCAS) and ***paratypes*** 15♂ 19♀ (IZCAS) **China**: Yunnan Province, Mengla County, Menglun Town, Rubber Plantation near Menglun Nature Reserve (21.90000°N, 101.26667°E; 569 m alt.), 1–15.V.2007, G. Zheng leg.; 2♂ 2♀ (IZCAS): Yunnan Province, Mengla County, Menglun Town, Menglun Nature Reserve, rubber-tea plantation (21.91667°N, 101.26667°E; 572 m alt.), 5–12.I.2007, G. Zheng leg.; 2♀ (IZCAS) same locality, secondary seasonal tropical rainforest (21.90000°N, 101.28333°E; 612 m alt.), 10.VIII.2007, G. Zheng leg.

##### Other material examined.

1♂ 1♀ (NHMSU-HA082) **China**: Yunnan Province, Mengla County, Menglun Town, Xishuangbanna Tropical Botanic Garden, in forest of *Paramicheliabaillonii* (21.91207°N, 101.26836°E; 527 m alt.), 2.X.2017, Y. Lin and Y. Li leg.; 1♂ (NHMSU-HA082) and 1♀ (NHMSU-HA082) used for sequencing, GenBank: MW970238 and MW970237, same data as for preceding; 1♂ (NHMSU-HA060): Yunnan Province, Mengla County, Menglun Town, Xishuangbanna Tropical Botanic Garden, Rubber-Tea plantation (21.91077°N, 101.27095°E, 572 m alt.), 8–12.VIII.2006, G. Zheng leg.; 1♀ (NHMSU-HA076): Yunnan Province, Xishuangbanna Natural Reserve, monsoon forest off greenstone road, in the bamboo forest (21.90707°N, 101.28183°E, 607 m alt.), 24.V.2013, Z. Zhao and Z. Chen leg.

##### Diagnosis.

see diagnosis for *S.longtou.*

##### Description.

**Male** (NHMSU-HA082). Total length 0.52. Carapace 0.28 long, 0.32 wide, 0.32 high. Clypeus 0.14 high. Sternum 0.20 long, 0.20 wide. Abdomen 0.40 long, 0.32 wide, 0.36 high. Length of legs: I 1.00 (0.28, 0.12, 0.24, 0.16, 0.20); II 0.78 (0.18, 0.12, 0.18, 0.12, 0.18); III 0.58 (0.12, 0.08, 0.12, 0.10, 0.16); IV 0.80 (0.20, 0.12, 0.18, 0.12, 0.18).

**Somatic characters** (Fig. 21A–C). ***Colouration***: carapace brown, sternum light brown ventrally. Legs light brown. Abdomen pale at middle, light brown laterally and ventrally. ***Prosoma***: PME separated by ~ ⅓ their diameter. ALE protruded, PER slightly recurved. Clypeus concave, smooth. Sternum surface rugose, pitted, slightly plump. ***Legs***: with long disto-dorsal spine on patella; 2 long dorsal spines on tibiae I and II, 1 on tibia III and IV. ***Opisthosoma***: round dorsally, ovoid laterally, extended posteriorly beyond spinnerets, abdominal surface with sparse, long setae, with a postero-lateral scutum. Spinnerets light brown, surrounded by an annular plate.

***Palp*** (Fig. 22A and B): bulb oblate, femur plump. Cymbium broad, with needle-like apical process and nodular distal one. Tegulum nearly rectangular. Median apophysis small, tubercle-like. Conductor long, wide basally, narrow mesally and distally. Embolus long, jutting out from prolateral margin of tegulum, curved upwards, extended beneath distal part of cymbium.

**Female** (NHMSU-HA076). Total length 0.80. Carapace 0.32 long, 0.32 wide, 0.28 high. Clypeus 0.12 high. Sternum 0.20 long, 0.20 wide. Abdomen 0.56 long, 0.56 wide, 0.60 high. Length of legs: I 0.84 (0.24, 0.12, 0.20, 0.12, 0.16); II 0.72 (0.18, 0.10, 0.16, 0.12, 0.16); III 0.60 (0.16, 0.10, 0.12, 0.10, 0.12); IV 0.78 (0.20, 0.10, 0.20, 0.12, 0.16).

**Somatic characters** (Fig. 21D–F). ***Colouration***: prosoma and legs as in male. Abdomen pale dorsally and light grey ventrally. Carapace modified as in male. Cephalic part lower than in male. ***Legs***: the spination same as in male, except tibia II lacking clasping spine. ***Opisthosoma***: globose, with sparse, long setae, without posterolateral scutum. Other modifications same as in male.

***Epigyne*** (Fig. 22C–E): sclerotised, cuticle weakly rugose. Epigynal posteromargin slightly protruded. Spermathecae separated by ca. 1.5 diameters. Copulatory openings adjacent. Copulatory ducts long, encircle spermathecae, forming ~ ¾ loop from posterior to anterolateral connecting with the inner middle margins of spermathecae. Fertilisation ducts short, starting at postero-lateral margin of spermathecae, extending to lateral of posterior epigynal margin.

##### Distribution.

China (Yunnan) (Fig. 23).

## Discussion

The taxonomy of genus *Patu* is revised in the current study and the taxonomic positions of some puzzling Asian *Patu* species are resolved. However, the species here are only the “tip of the iceberg” of Asian *Patu* species (Yao et al. 2021, Li et al. 2021) and further studies are necessary to revise the worldwide *Patu* spiders.

## Supplementary Material

XML Treatment for Patu

XML Treatment for Patu catba

XML Treatment for Patu dakou

XML Treatment for Patu damtao

XML Treatment for Patu jiangzhou

XML Treatment for Patu jidanweishi

XML Treatment for Patu nagarat

XML Treatment for Patu nigeri

XML Treatment for Patu putao

XML Treatment for Patu qiqi

XML Treatment for Patu xiaoxiao

XML Treatment for Kirinua

XML Treatment for Kirinua maguai

XML Treatment for Kirinua yangshuo

XML Treatment for Swilda

XML Treatment for Swilda longtou

XML Treatment for Swilda spinathoraxi

## Appendix 1

**Table A1.** Uncorrected genetic pairwise distances (lower triangle) and standard errors (upper triangle) of a partial fragment of COI from ten species discussed in this text.

Species			1		2	3		4		5	6		7	8		9		10	
			♀	♂	♀	♀	♂	♀	♂	♀	♀	♂	♀	♀	♂J	♀	♂	♀	♂
1	*P.dakou* sp. nov.	♀		0.001	0.012	0.013	0.013	0.012	0.012	0.016	0.013	0.013	0.019	0.018	0.018	0.018	0.018	0.019	0.019
		♂	0.002		0.012	0.013	0.013	0.012	0.012	0.016	0.013	0.013	0.019	0.018	0.018	0.018	0.018	0.019	0.019
2	*P.jiangzhou* sp. nov.	♀	0.101	0.099		0.013	0.013	0.014	0.014	0.015	0.011	0.011	0.017	0.018	0.018	0.017	0.017	0.017	0.017
3	*P.jidanweishi*	♀	0.092	0.090	0.105		0.000	0.013	0.013	0.014	0.013	0.013	0.018	0.019	0.019	0.017	0.017	0.016	0.016
		♂	0.092	0.090	0.105	0.000		0.013	0.013	0.014	0.013	0.013	0.018	0.019	0.019	0.017	0.017	0.016	0.016
4	*P.nagarat* sp. nov.	♀	0.094	0.092	0.116	0.094	0.094		0.000	0.017	0.013	0.013	0.019	0.020	0.020	0.018	0.018	0.017	0.017
		♂	0.094	0.092	0.116	0.094	0.094	0.000		0.017	0.013	0.013	0.019	0.020	0.020	0.018	0.018	0.017	0.017
5	*P.nigeri*	♀	0.129	0.127	0.141	0.107	0.107	0.146	0.146		0.015	0.015	0.018	0.019	0.019	0.018	0.018	0.017	0.017
6	*P.xiaoxiao*	♀	0.092	0.090	0.073	0.092	0.092	0.099	0.099	0.121		0.002	0.017	0.019	0.019	0.016	0.016	0.017	0.017
		♂	0.094	0.092	0.075	0.094	0.094	0.101	0.101	0.123	0.002		0.016	0.019	0.019	0.016	0.016	0.017	0.017
7	*K.maguai* sp. nov.	♀	0.197	0.195	0.169	0.189	0.189	0.203	0.203	0.181	0.163	0.165		0.016	0.016	0.016	0.016	0.017	0.017
8	*K.yangshuo* sp. nov.	♀	0.191	0.189	0.185	0.191	0.191	0.212	0.212	0.202	0.193	0.195	0.142		0.000	0.018	0.018	0.019	0.019
		♂J	0.191	0.189	0.185	0.191	0.191	0.212	0.212	0.202	0.193	0.195	0.142	0.000		0.018	0.018	0.019	0.019
9	*S.longtou*	♀	0.187	0.185	0.161	0.165	0.165	0.183	0.183	0.177	0.155	0.157	0.146	0.187	0.187		0.000	0.013	0.013
		♂	0.187	0.185	0.161	0.165	0.165	0.183	0.183	0.177	0.155	0.157	0.146	0.187	0.187	0.000		0.013	0.013
10	*S.spinathoraxi*	♀	0.199	0.197	0.153	0.149	0.149	0.179	0.179	0.168	0.155	0.157	0.167	0.197	0.197	0.103	0.103		0.002
		♂	0.197	0.195	0.151	0.147	0.147	0.177	0.177	0.166	0.153	0.155	0.165	0.195	0.195	0.101	0.101	0.002	
